# Concurrent influence of top-down and bottom-up inputs on correlated activity of Macaque extrastriate neurons

**DOI:** 10.1038/s41467-018-07816-4

**Published:** 2018-12-19

**Authors:** Yaser Merrikhi, Kelsey Clark, Behrad Noudoost

**Affiliations:** 1 School of Cognitive Sciences, Institute for Research in Fundamental Sciences (IPM), Tehran, 193955746, Iran; 2 Department of Ophthalmology and Visual Sciences, University of Utah, Salt Lake City, UT 84132 USA

## Abstract

Correlations between neurons can profoundly impact the information encoding capacity of a neural population. We studied how maintenance of visuospatial information affects correlated activity in visual areas by recording the activity of neurons in visual area MT of rhesus macaques during a spatial working memory task. Correlations between MT neurons depended upon the spatial overlap between neurons’ receptive fields. These correlations were influenced by the content of working memory, but the effect of a top-down memory signal differed in the presence or absence of bottom-up visual input. Neurons representing the same area of space showed increased correlations when remembering a location in their receptive fields in the absence of visual input, but decreased correlations in the presence of a visual stimulus. This set of results reveals the correlating nature of top-down signals influencing visual areas and uncovers how such a correlating signal, in interaction with bottom-up information, could enhance sensory representations.

## Abstract (web summary)

Changes in correlated activity of neurons are believed to influence their information coding capacity. Here, the authors show how top-down and bottom-up inputs and their interaction differentially alter the correlated activity of neurons in extrastriate cortex

## Introduction

One of the unique features of how the brain processes and represents information is its massive parallelization: many neurons simultaneously contribute to the representation of a single stimulus. This means that the trial-by-trial correlations between neurons will play an important role in the population representation^1–4^, and some of these relationships can only be understood by simultaneously recording the activity of multiple neurons.

Sampling the pairwise correlations between simultaneously recorded neurons (noise correlations) can give important insights into the population-level encoding^5–8^. Modulating noise correlations is believed to be a means for enhancing representations. Indeed, spatial attention decorrelates neurons in a way that enhances the population representation^9, 10^. Attention studies have looked at neurons receiving diverse top–down signals, and reported both correlation and decorrelation effects^11^; indeed, fluctuations in the attentional state have also been shown to contribute to cross-trial variability^12, 13^. Our recent work^14, 15^ has shown that during spatial working memory (WM), extrastriate visual areas receive a top–down WM signal from the prefrontal cortex (PFC) and undergo subthreshold modulations based on the content of WM (see Supplementary Note 1 for more details).

In order to better understand the influence of top–down signals on the representation of information within sensory areas we studied how a top–down spatial signal modulates the correlated activity of pairs of neurons within extrastriate areas, and how these modulations depend on the similarity between the spatial and feature selectivity of the two neurons and the locus of spatial WM. To test whether the maintenance of spatial information alters the correlated variability of these neurons, we recorded their activity during the memory guided saccade (MGS) task, in the presence or absence of a visual stimulus during the memory period. This systematic investigation revealed that an isolated spatial signal increases correlations between neurons with similar RFs; however, in the presence of a visual probe the top–down spatial signal instead causes a decorrelation of their responses, consistent with the results of attention studies. For neurons with less similar RFs, a top–down spatial signal in isolation decreases the correlation between neurons, but in the presence of a visual stimulus this top–down signal increases their correlation. These results suggest an excitatory, correlating role for a top–down signal received by visual areas in isolation, and highlight the significance of interactions between bottom–up and top–down signals in determining the correlated activity of neurons in these areas. Based on the experimental data, we present a descriptive network model to elucidate how the top–down and bottom–up signals interact to generate the observed patterns of correlated variability among extrastriate cortical neurons.

## Results

### Baseline noise correlations depend upon RF similarity

**Fig. 1:**
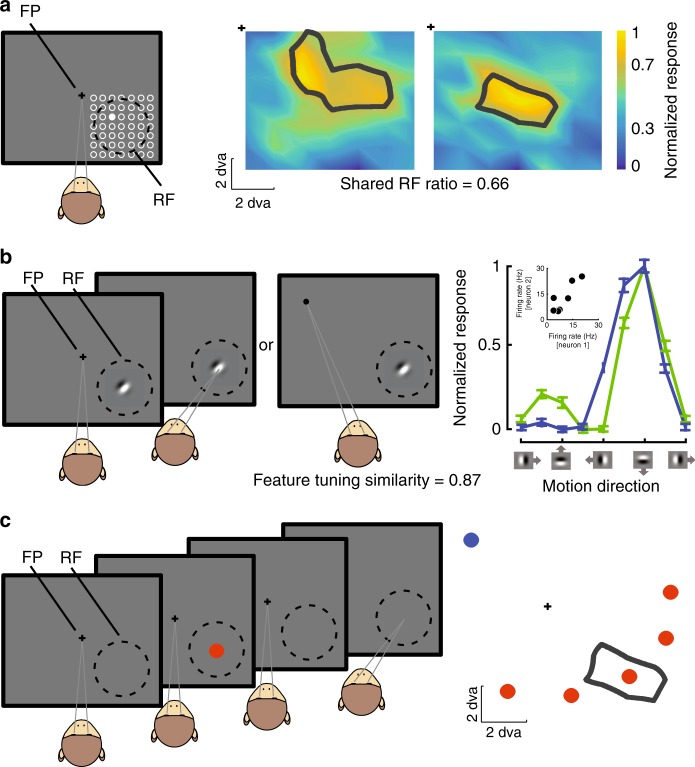
Paradigms for measuring spatial RF, feature tuning, and WM modulation. **a** Spatial RF mapping during fixation and quantifying RF overlap between neurons. In the RF mapping task, the monkey fixates in the center of the screen while probes flash in a grid covering the estimated RF location. For each neuron, an RF profile is constructed based on locations in which probe-evoked activity is at least 50% of the maximum probe-evoked response. The RF overlap between two neurons is measured using the formulas in the Methods: Data analysis (the sample pair’s shared RF ratio = 0.66). **b** Visually guided saccade task for measuring motion direction tuning. The monkey fixates and a moving grating stimulus appears in the neuron’s RF; the grating can be moving in one of eight directions. When the fixation point disappears, the monkey saccades either to the grating stimulus, or to a saccade target which appears in the opposite visual hemifield. To calculate the similarity in feature tuning between two neurons, we measured the correlation coefficient for the average firing rate of the two neurons in response to the eight motion directions (Tuning curves of the same sample pair to the right; inset scatter plot, Pearson correlation coefficient = 0.87). **c** Memory guided saccade task. The monkey fixates, and a cue stimulus appears in one of six positions arranged around the neuron’s RF location. The cue stimulus disappears, and the monkey maintains fixation throughout a blank delay period. Following the disappearance of the fixation point, the monkey saccades to the remembered location to receive a reward. An example MT neuron’s RF (black contour) and the relative positioning of the MGS target locations (gray dots) are shown to the right; we frequently compare activity or correlations during memory IN condition (red dots) vs. memory OUT (blue dot). Cross indicates fixation point

Spiking activity was recorded from 85 neurons in the MGS task (350 pairs over 14 recording sessions) and 114 neurons in the MGS task with visual probes (358 pairs over 20 recording sessions) in area MT of two monkeys using array electrodes. We measured each neuron’s spatial RF, feature tuning curve, and WM-related modulations using three different tasks (Fig. 1). Spatial RFs were measured using the neuron’s response to a grid of probes presented during fixation (Fig. 1a), and motion sensitivity was assessed based on the response to moving gratings (Fig. 1b). For each pair of neurons, we quantified the spatial overlap in their RFs based on the proportion of the RF area falling within both RF contours (see the formula (1) in Methods: Data analysis), and the similarity of their feature tuning based on the correlation coefficient for the responses of the two neurons to different motion directions (Fig. 1b). To assess the noise correlation, the trial-by-trial correlation in responses of pairs of simultaneously recorded neurons was measured. Knowing the shared spatial and feature similarity, we then tested the response of pairs of neurons during the delay period of an MGS task as the remembered location varied relative to the neurons’ RFs (Fig. 1c and Methods: Behavioral tasks). The animal performed the MGS task at a total of 6 locations, 5 close to the measured RFs of the simultaneously recorded MT neurons and one in the opposite hemifield. This arrangement enabled us to systematically investigate the effects of WM on neurons with various degrees of RF overlap with the memorized location.

First, we examined the degree to which noise correlations depend upon the feature and spatial tuning similarity between pairs of neurons during the fixation period of the MGS task. Consistent with previous findings^16^, the noise correlation between neurons depends upon the overlap between their spatial RFs (*r* = 0.120, *p* < 10^−8^, linear regression), but is independent of the similarity in their feature tuning (*r* = 0.008, *p* = 0.663, linear regression; no significant interaction between spatial and feature tuning: *r* = 0.037, *p* = 0.394, linear regression) (Supplementary Figure 1). RF overlap was a better predictor of noise correlations than physical distance between neurons (Supplementary Note 2, Supplementary Figure 2). Based on this primary dependence on spatial, rather than feature similarity, we focused on how a top–down spatial signal alters correlations between pairs of neurons with varying degrees of spatial similarity. We measured the activity and correlations of MT neurons during the delay period of the MGS task (in the absence of any visual stimuli). Consistent with previous reports that MT and other early visual areas lack persistent activity in WM tasks^17–19^, we also confirmed that the firing rates of MT neurons during the delay period do not reflect the location held in WM. There was no significant change in the delay period firing rate based on whether the location held in WM was in the same (memory IN) or the opposite (memory OUT) hemifield from the neuron’s RF, either for single units (*n* = 85, ∆FR_IN–OUT_ = −0.22 ± 0.15 Hz, *p* = 0.656, mean ± s.e.m) or for multi-unit activity (*n* = 122, ∆FR_IN–OUT_ = −0.07 ± 0.12 Hz, *p* = 0.748). We also confirmed that the firing rate does not reflect the locus of WM by comparing the firing rate between the five conditions for which the memory location is in the same hemifield as the neurons’ RFs (*F* = 0.03, *p* = 0.998, One-way ANOVA, Supplementary Figure 3). Given that firing rate did not change between the five memory conditions, we expected noise correlations to remain constant as well. Indeed, quantifying the correlations between pairs of neurons separately for each condition, we did not find any effect of WM location on the mean noise correlation of all the simultaneously recorded pairs of MT neurons (*F* = 0.32, *p* = 0.863, One-way ANOVA). We next grouped the five memory IN conditions and compared the noise correlation in these conditions to memory OUT. There was no significant change in the mean noise correlation for memory IN vs. OUT (F = 0.16, *p* = 0.687, One-way ANOVA, ∆correlation_IN–OUT_ = −0.002 ± 0.002, *p* = 0.482, *n* = 350, mean ± s.e.m). Thus, looking at the overall firing rate and noise correlation, we did not find any reflection of the location held in WM in the activity of MT neurons.

### WM-induced noise correlations depend on neurons' RF overlap

**Fig. 2:**
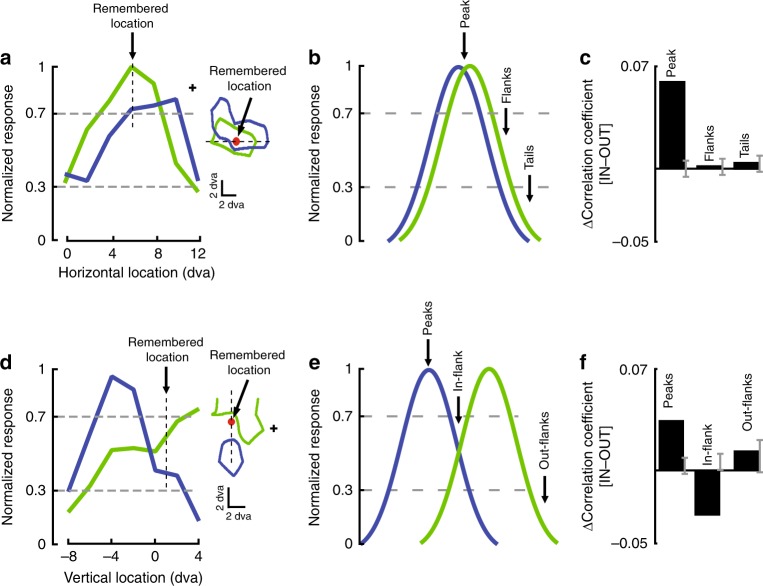
Changes in noise correlations during WM depend upon RF overlap and memory location. **a** RF profiles for a pair of example neurons with overlapping RFs. (Left) Firing rate as a function of horizontal location in visual space. The vertical dashed line indicates the remembered location. (Right) RF profiles plotted as the 70%-of-max contour in two dimensions. The dashed line indicates the slice used for the 1-dimensional plot to its left, the red dot indicates the remembered location, and the cross indicates fixation point. **b** A schematic illustration of the spatial RF profiles and WM locations for an overlapping pair of neurons. WM targets could appear near the peak of both RF profiles (>0.7, Peak), in an intermediate portion of both RFs (0.3–0.7, Flanks), or in an area with little response (<0.3, Tails). Horizontal dashed lines indicate 30% and 70% of maximum normalized firing rate. **c** WM-induced changes in noise correlations for the same example pair of neurons shown in A. We compared the noise correlation for targets in or near the RFs to those appearing in the opposite hemifield (IN–OUT). For this pair of spatially similar neurons, the noise correlation was greater when remembering a Peak location compared to a location outside both (*p* = 2×10^−9^), but remembering a location in the Flanks or Tails of the profile had no effect (Flanks, *p* = 0.912; Tails, *p* = 0.378, Wilcoxon signed-rank test compared to shuffled data; gray bars show SEM for shuffled data). **d** RF profiles for a pair of example neurons with partially overlapping RFs. Conventions as in (**a**). **e** A schematic illustration of the RF profiles and WM locations for a partially overlapping pair. WM targets could appear at the peak of one neuron’s RF but outside the other neuron’s RF (Peaks), at a location with 0.3–0.7 response from both neurons (In-flank), or at a location with only some response from one neuron (Out-flanks). **f** WM-induced changes in noise correlations (IN–OUT) for the example pair in (**d**). For this pair of partially overlapping neurons, noise correlation decreased when remembering In-flank locations (*p* = 1×10^−8^); Peaks and Out-flanks memory locations became slightly more correlated (Peaks *p* = 7×10^−6^; Out-flanks *p* = 0.533)

The initial analysis did not show changes in average noise correlation based on the remembered location. However, recent findings by Wimmer and colleagues suggest that the noise correlation should be altered in different ways based on the spatial relationship between pairs of neurons^20^. These authors reported that during spatial WM, noise correlations between pairs of neurons within the PFC are positive for neurons with similar spatial tuning when the remembered location is at the peak of two neurons' RFs, but negative for remembered locations between two neurons’ RF peaks with dissimilar spatial tuning, consistent with a moving peak of activity in a bump attractor model of memory maintenance. Given that prefrontal projections to visual cortex contain information about the content of spatial WM^14^, we predicted that a similar dependence of shared variability on the similarity of spatial tuning and memory location would be evident in MT responses. Indeed, splitting neuron pairs based on their shared spatial sensitivity revealed that the ability of a WM signal to alter the correlation between a pair of neurons depends on the degree to which those neurons are sensitive to the location held in WM. Figure 2 illustrates the effect of WM on the correlations between two example MT pairs with differing degrees of similarity in their spatial sensitivity. The inset in Fig. 2a shows the contour of the RFs for two simultaneously recorded MT neurons. These are the same two neurons shown in Fig. 1, with a shared RF ratio of 0.66. As shown, the WM location is within the RF of both of these neurons. Figure 2a shows the RF profiles of these neurons (the cross-section of the RF along the shared dimension, *x* axis in this case), indicating that the WM target is presented near the peak of both neurons’ RFs. For a pair of neurons like this, with very similar RF profiles (overlapping pairs), the different WM locations will either fall within the Peak (both responses >70% of maximum), Flanks (both responses 30–70% of maximum), or Tails (both responses <30% of maximum) of the RF profiles, as schematically illustrated in Fig. 2b. The effect of WM on the correlation between this pair of neurons depends on whether the WM location is in the Peak, Flanks, or Tails location. Even though the firing rate is not altered by WM, we still performed a rate-matching control when calculating the correlation values (see Methods: Data analysis). All correlation changes are reported as the change compared to the Memory OUT condition. For this pair of MT neurons we observed a significant increase in the correlation between their responses when the locus of WM is at their Peak compared to memory OUT (∆correlation_Peak_ = 0.060, *p* = 2×10^−9^, Wilcoxon signed-rank test compared to shuffled data), whereas no significant change in correlations occurs when remembering a location in the Flanks or Tails of the RF profiles (∆correlation_Flanks_ = 0.002, *p* = 0.912; ∆correlation_Tails_ = 0.005, *p* = 0.378, Wilcoxon signed-rank test compared to shuffled data) (Fig. 2c).

The effect of WM on the correlations between neurons was different for pairs whose RF profiles were only partially overlapping, as illustrated in Fig. 2d–f. Figure 2d shows the RF contours for an example partially overlapping pair, and Fig. 2e schematically shows the potential WM locations relative to the neurons’ RF profiles. For pairs of neurons with partially overlapping RFs, we refer to the potential memory locations as Peaks (>70% for one neuron), In-flank (30–70% for both neurons), and Out-flanks (<30% for both neurons). For this sample pair we observed a reduction in the noise correlation for the In-flank condition and an increase for the Peaks condition, compared to when remembering a location in the opposite hemifield (∆correlation_In-flank_ = −0.032, *p* = 1×10^−8^, ∆correlation_Peaks_ = 0.035, *p* = 7×10^−6^, Wilcoxon signed-rank test compared to shuffled data). For this example pair, we did not observe any significant change in the noise correlation when the WM target is presented in the Out-flanks (∆correlation_Out-flanks_ = 0.014, p = 0.533, Wilcoxon signed-rank test compared to shuffled data) (Fig. 2f).

**Fig. 3:**
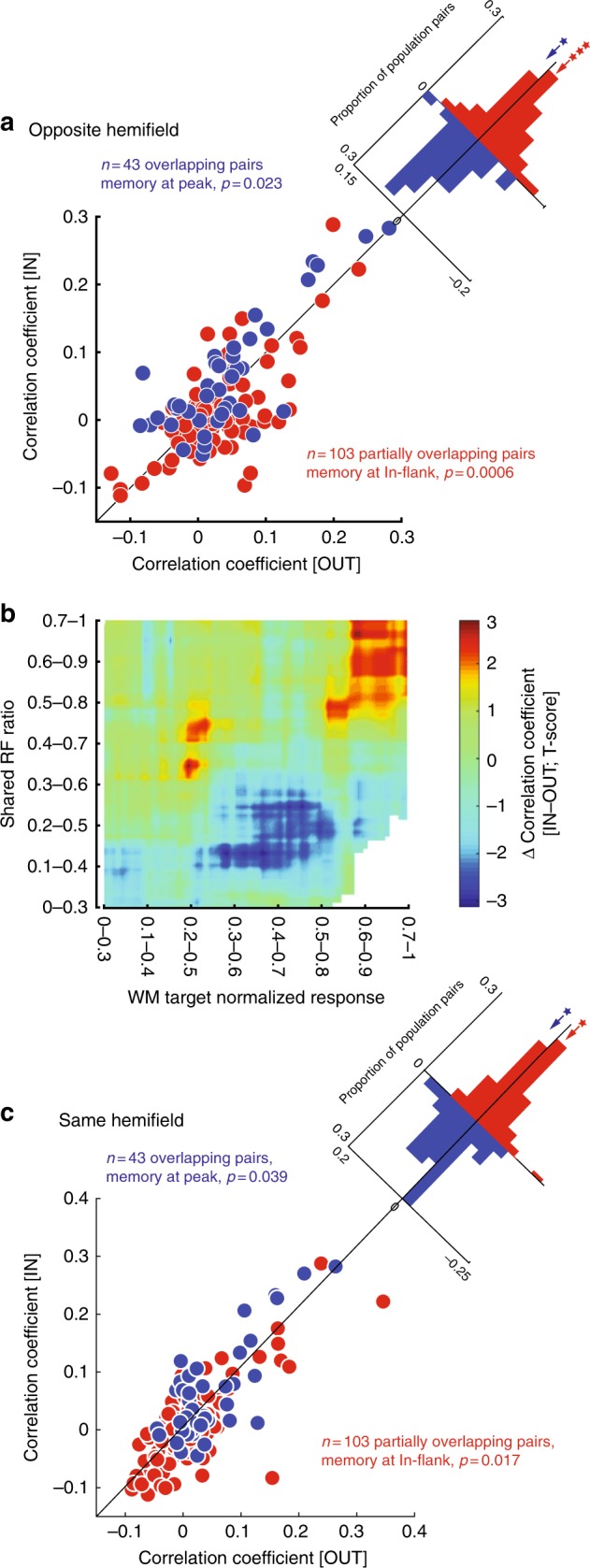
Population changes in noise correlation as a function of RF overlap and target location. **a** Population-level changes in noise correlations for overlapping and partially overlapping pairs of neurons. For pairs of neurons with overlapping RFs, remembering a stimulus at the Peak location increased the noise correlation relative to a location in the opposite hemifield (blue; *n* = 43, *p* = 0.023). For pairs of neurons with partially overlapping RFs, remembering an In-flank location decreased the noise correlation relative to a location in the opposite hemifield (red; *n* = 103, *p* = 0.0006). **b** Heatmap shows the change in noise correlation (IN–OUT condition) for pairs of neurons sorted based on their RF overlap (*y*-axis) and the memory location relative to their maximal responses. This is a continuous plot of the data grouped into overlapping and partially overlapping populations in (**a**). Memory location is plotted relative to the normalized response level of the neurons at that location. Data on both axes were binned in units of 0.3. The main effects are visible here: for overlapping neurons (shared RF ratio>0.5), target locations near the peak of both RFs (high *x*-axis value, corresponding to Peak designation in Fig. 2b) show increased correlation. For partially overlapping neurons (0.1<shared RF ratio< 0.4), target locations with intermediate responses from both neurons (middle *x*-axis values, corresponding to In-flank designation in Fig. 2e) show decorrelation. **c** Population-level changes in noise correlations for overlapping and partially overlapping pairs of neurons. Same as (**a**), but comparing memory IN to a memory location outside both neurons’ RFs but in the same visual hemifield. For pairs of neurons with overlapping RFs, remembering a stimulus at the Peak location increased the noise correlation relative to a location outside both RFs (blue; *n* = 43, *p* = 0.039). For pairs of neurons with partially overlapping RFs, remembering an In-flank location decreased the noise correlation relative to a location outside both RFs (red; *n* = 103, *p* = 0.017)

At the population level two main effects were consistently observed: an increased correlation between overlapping pairs when remembering a Peak location, and a decorrelation of partially overlapping pairs when remembering an In-flank location. For pairs with overlapping RFs, remembering a Peak RF location increased their noise correlation during the delay period from 0.053 ± 0.016 (mean ± s.e.m) when remembering a location in the opposite hemifield to 0.069 ± 0.016 when remembering a location at the Peak (*n* = 43 pairs, ∆correlationPeak = 0.016 ± 0.008, *p* = 0.023; Fig. 3a, blue). For pairs of neurons with partially overlapping RFs, remembering an In-flank location decorrelated their activity, decreasing the average correlation coefficient from 0.025 ± 0.006 when remembering a location in the opposite hemifield to 0.008 ± 0.006 when remembering an In-flank location (*n* = 103 pairs, ∆correlation In-flank = −0.017 ± 0.005, *p* = 6×10^−4^; Fig. 3a, red). No other combinations of RF configuration and memory location showed changes in noise correlation during WM (Supplementary Notes 3&4, Supplementary Figure 4). Microsaccades did not correlate with changes in noise correlations, nor did excluding the period after microsaccades from the analysis alter the significance of the main findings (Supplementary Note 5, Supplementary Figure 5). Therefore, WM increases the correlations between neurons when remembering a location at the Peak of overlapping pairs, and reduces correlations when remembering an In-flank location for partially overlapping pairs. This set of results suggests that, as expected for an excitatory common input, a pure WM signal correlates the activity of neurons receiving the same spatially selective signal, and decorrelates neurons which receive signals with different spatial profiles.

The excitatory, correlating effect of memory maintenance is illustrated in a different way in Fig. 3b. Rather than grouping pairs and WM locations, the changes in correlations between pairs are quantified as a function of continuous values for the degree of RF overlap and the WM location. The change in the noise correlation was measured for memory OUT vs. memory IN, with the distance of the memory location from the RF peaks shown on the *x*-axis. Therefore, each pair may contribute to this graph more than once; for example, for a hypothetical pair of completely overlapping neurons, when the WM locus is exactly at their peak the value of their change in noise correlation will be reflected in the top-right corner of the plot, and for conditions when the memory location is outside of both RFs it will be represented in the top-left corner. Consistent with Fig. 3a, when WM is at the peak (abscissa > 0.7) of two overlapping neurons (ordinate > 0.5) the correlation between neurons increases. For partially overlapping pairs (0.1 < ordinate < 0.4), a reduced correlation is observed when the WM locus is In-flank for both neurons (0.3 < abscissa < 0.7). Consistent with previous analysis, WM did not strongly modulate the noise correlation in other arrangements.

The same main effects (an increase in the correlation of overlapping pairs when remembering a Peak location, a decrease in the correlation of partially overlapping pairs when remembering an In-flank location) were seen when comparing Peak or In-flank memory conditions to a memory cue outside both neurons’ RFs, but within the same visual hemifield. For overlapping RF pairs, remembering a Peak location increases the correlated variability of neurons compared to a location in same hemifield but outside of both RFs (*n* = 43 pairs, ∆correlation_Peak_ = 0.019 ± 0.009, *p* = 0.039; Fig. 3c, blue). For partially overlapping RF pairs, remembering an In-flank location decreases the correlated variability of neurons compared to a location in same hemifield but outside of both RFs (*n* = 103 pairs, ∆correlation_In-flank_ = −0.012 ± 0.005, *p* = 0.017; Fig. 3c, red). Moreover, the effects for same-hemifield comparisons are not significantly different in magnitude than those observed for the opposite hemifield comparisons (∆correlation_Peak_ = 0.000 ± 0.008, *p* = 0.771, *n* = 43 pairs; ∆correlation_In-flank_ = 0.006 ± 0.006, *p* = 0.128, *n* = 103 pairs). This control analysis reemphasizes the spatial dependence of changes in correlations, and suggests that the spatial extent of changes in correlated activity is probably on the order of RF size.

### Top–down and bottom–up signals determine noise correlations

**Fig. 4:**
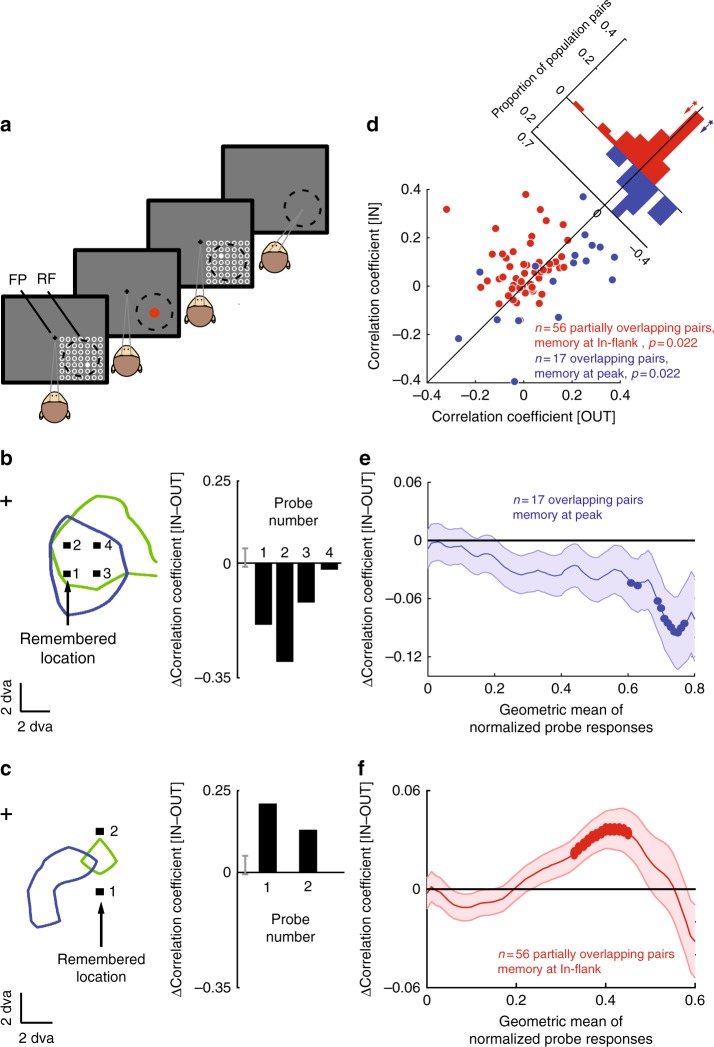
Changes in noise correlations of visual responses during WM. **a** MGS task with visual probes. The task was the same as the regular MGS task, except that brief (200 ms) visual probes flashed on screen during both the fixation and delay periods. **b** Changes in noise correlations of visual responses for an example pair of neurons with overlapping RFs. RF profiles (green and blue), probes (black squares), and WM location are shown in visual space relative to the fixation point (cross). All four probes and WM locations were presented at the Peak location. Bar graphs indicate change in noise correlation (IN–OUT) for the four visual probe locations; for all four probe locations shown, the noise correlation decreased during memory IN. **c** Changes in noise correlations of visual responses for an example pair of neurons with partially overlapping RFs. Conventions as in **b**. The two probes and WM locations were presented at the In-flank location. Bar graphs indicate change in noise correlation (IN–OUT) for the two visual probe locations: the noise correlation increased during memory IN compared to OUT. **d** For neurons with overlapping RFs (blue), noise correlations of visual responses decreased when remembering a Peak location (*n* = 17 pairs, *p* = 0.022). For neurons with partially overlapping RFs (red), noise correlations of the visual responses increased when remembering an In-flank location (*n* = 56 pairs, *p* = 0.022). **e** Changes in noise correlation as a function of visual probe location: for overlapping pairs of neurons, remembering a target in the Peak location decreases the noise correlation for probes appearing at Peak RF locations. Probe location (*x*-axis) here and in (**f**) is plotted according to the geometric mean of the two normalized RF profile responses at that location $\left( {\sqrt {{\rm{RF1}}\,{\rm{response}} \ast {\rm{RF2}}\,{\rm{response}}} } \right)$. Neuron pairs are plotted in a 0.3 wide bin (*x*-axis); areas in bold had a significant effect of memory location on noise correlation (*p* < 0.05). **f** Changes in noise correlation as a function of visual probe location: for partially overlapping pairs of neurons, remembering a target in the In-flank location increases the noise correlation for probes appearing at In-flank RF locations

This finding of an increased correlation between neurons with overlapping RFs receiving a common spatial signal seems to conflict with previous reports of decorrelation during spatial attention^9, 11^. Since one clear difference between our WM paradigm and attention tasks was the presence of a visual stimulus, we next sought to directly test the contribution of a top–down signal to the correlations between extrastriate neurons in the presence of a visual signal. To do this, we used a task in which brief visual probes were presented both during fixation and during the memory period of the MGS task (Fig. 4a). First, we verified that the visual probes did not alter overall memory performance (Supplementary Note 6, Supplementary Figure 6). We then compared the noise correlations for visually evoked responses during the delay period across the memory conditions. Interestingly, the presence of a visual stimulus dramatically changes the contribution of a WM signal to noise correlations; indeed, it reverses most of the effects observed for a WM signal alone. In the presence of a visual stimulus, pairs of neurons with overlapping RFs were decorrelated when remembering the Peak location. The RF arrangement of an example pair of neurons is shown in Fig. 4b. The arrow indicates the locus of WM, and the black squares show the location of four visual probes presented during the memory period inside the overlapping portion of the two neurons’ RFs. The animal had to remember a location, which happened to match one of the probe locations in this case (probe 1). Using the same definition as before, therefore, the WM locus was at the Peak location. The bar graph shows the noise correlation for this pair of neurons, for each of the four example visual probes during this Peak condition compared to the memory OUT condition. We observed a reduction in the noise correlation (average of ∆correlation_Peak_ across four visual probes = −0.158, *p* = 2×10^−6^, Wilcoxon signed-rank test compared to shuffled data). Therefore, in contrast to the WM-induced increase in noise correlation observed for overlapping pairs in the absence of a visual stimulus, for this example overlapping pair, a WM signal decorrelates their activity in the presence of visual input. We observed a similar change in the direction of the WM effect for partially overlapping pairs of neurons. Figure 4c illustrates the locus of WM, the location of visual probes, and the RF arrangement for an example pair of neurons with partially overlapping RFs. As shown in the bar graph, for this pair, an In-flank memory location increases the correlation between the responses to both In-flank probes (average ∆correlation_In-flank_ for both visual probes = 0.175, *p* = 6×10^−9^ for both visual probes, Wilcoxon signed-rank test compared to shuffled data). Thus, the effects of WM on noise correlations are reversed in the presence of visual stimuli: for pairs of neurons with overlapping RFs, remembering the Peak location increases correlations in isolation but decreases correlations in the presence of visual stimuli; for pairs of neurons with only partially overlapping RFs, remembering an In-flank location decreases correlations in isolation but increases correlations in the presence of visual stimuli.

This reversal in the effect of WM on noise correlations in the presence of visual input was observed across the population. In the presence of visual stimuli, WM decorrelated the memory period responses for pairs of neurons with overlapping RFs, with the mean correlation decreasing from 0.131 ± 0.047 when remembering a location in the opposite hemifield, to 0.030 ± 0.045 for memory at the Peak (Fig. 4d, blue; *n* = 17 pairs, ∆correlation_Peak_ = −0.102 ± 0.038, *p* = 0.021). The increased correlation for the In-flank condition of partially overlapping pairs was also observed across the population, with the average correlation increasing from 0.011 ± 0.013 when remembering a location in the opposite hemifield, to 0.068 ± 0.014 when remembering an In-flank location (Fig. 4d, red; *n* = 56 pairs, ∆correlation_In-flank_ = 0.057 ± 0.018, *p* = 0.022). No other combinations of RF configuration and memory location showed changes in noise correlations during WM in the presence of visual probes (Supplementary Notes 3&4, Supplementary Figure 7). Therefore, for probes presented at the locus of WM, the addition of a WM signal at the Peak location decorrelates pairs of neurons with overlapping RFs, while a WM signal at the In-flank location correlates pairs of neurons with partially overlapping RFs.

In order to further quantify the decorrelating effect of the WM signal in the presence of visual input, we examined the effect of a WM signal on probes in other locations relative to the WM locus. We quantified each probe position relative to the RFs of a neuron pair by measuring the response that the probe generates in both neurons, calculating the geometric mean of these values, and normalizing between 0 and 1. Thus, a value of 1 indicates a probe located at the peak of a completely overlapping pair of RFs. Pooling all probes presented to all overlapping pairs, we examined the change in the noise correlation (WM at Peak vs. WM in opposite hemifield) as a function of the mean probe-evoked response. For pairs with overlapping RFs, there was only a significant change in the noise correlation for probe locations near the Peak (Fig. 4e), corresponding to the WM location. For partially overlapping pairs and an In-flank WM location (Fig. 4f), the main effect was an increased correlation when probes generated a mid-range response. These effects confirm the previous findings: a reduced correlation when probes & WM locus are at the Peak of overlapping pairs, and an increased correlation when probes & WM locus are In-flank for partially overlapping pairs (Fig. 4b–f, Supplementary Figures 8&9). We also verified that prior stimulus history (adaptation) did not account for the observed changes in noise correlations (Supplementary Note 7, Supplementary Figure 10).

### The noise correlation effects are not due to RF changes

Previous work in our lab has shown that during WM, the RFs of neurons in MT shift and expand toward the remembered location^14^. This could alter the amount of RF overlap between partially overlapping pairs: for example, pairs of neurons that both shift toward the WM locus may overlap more during WM as a result of these RF changes, while pairs of neurons in which only one neuron’s RF shifts may overlap less. Given the dependence of noise correlations on RF overlap (Supplementary Figure 1), one possibility is that changes in the RFs could affect the correlated activity of neurons. We sought to determine whether such changes in RF overlap were responsible for the changes in correlations we observed during WM. First, we verified that the same types of RF changes during WM were apparent in the current dataset. In this dataset, there are 59 simultaneously recorded MT neurons with a well-shaped RF contour during the three memory IN conditions. For this set of neurons, during memory maintenance, the RF of neurons shifted (*n* = 59, ∆RF distance_memory-fixation_ = −0.230 ± 0.067 d.v.a., *p* = 0.002) and expanded (∆RF size_memory-fixation_ = 0.288 ± 0.131 d.v.a., *p* = 0.005) toward the remembered location (Supplementary Figure 11). The gain of visual responses also increased (∆max response_memory-fixation_ = 3.239 ± 0.450 Hz, *p* < 10^−6^). These findings are consistent with our own previous report^14^.

**Fig. 5:**
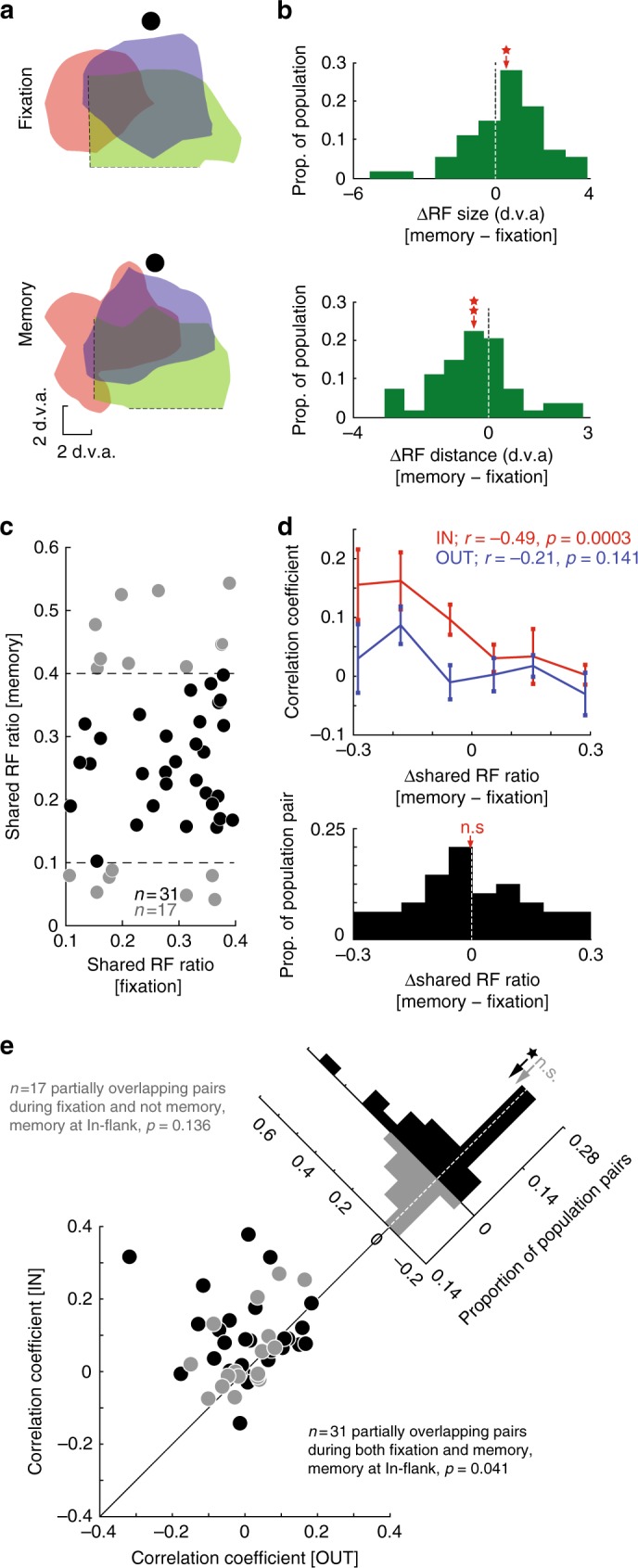
RF changes during WM do not account for changes in noise correlations. **a** RFs of three example neurons during fixation and memory. The RFs of two neurons (red and blue) expand and shift toward the remembered location, increasing their RF overlap. The RF of the third neuron (green) is mostly unchanged during WM, resulting in a decrease in overlap with the other neurons during WM. **b** During memory, RFs expand and shift toward the remembered location. Top histogram shows the distribution of changes in RF size during the memory period. RFs expanded during memory (*n* = 51 neurons, *p* = 0.014). Bottom histogram shows the distribution of changes in the distance of the RF center from the memory location. RFs shifted toward the remembered location (*n* = 51, *p* = 0.006). **c** RF overlap during fixation and memory. For pairs identified as partially overlapping (ratio of shared RF 0.1–0.4) during fixation, plot shows the ratio of shared RF during fixation and memory. **d** Noise correlation as a function of change in RF overlap. For partially overlapping pairs, the top plot shows the noise correlation during memory IN (red) and OUT (blue) as a function of the change in RF overlap. Pairs which experience a decrease in RF overlap during memory have the highest noise correlations during memory IN (*r* = −0.49, *p* = 0.0003). There was no significant relationship between change in RF overlap and noise correlation for memory OUT (*r* = −0.23, *p* = 0.116). Bottom histogram shows the distribution of changes in RF overlap across pairs. **e** Change in noise correlation of partially overlapping pairs during In-flank memory. Plot shows noise correlation during memory IN vs. OUT, for pairs of neurons meeting the definition of partially overlapping RFs during fixation and memory (black, *n* = 31), or only during fixation (gray, *n* = 17). Noise correlations increased during In-flank memory for pairs with partially overlapping RFs during both fixation and memory (*n* = 31 pairs, *p* = 0.041). There was no significant change in noise correlation for the pairs of neurons with partially overlapping RFs only during fixation (*n* = 17 pairs, *p* = 0.136). Histograms in the upper right show the distributions of change in noise correlation across pairs

In order to examine the relationship between changes in RF overlap and changes in correlated activity, we focused on pairs of neurons with partially overlapping RFs during the fixation period, since RF changes are more prominent when the WM locus is slightly off the center of the RF^14^. Fig. 5a shows an example of how three MT neurons change their RF profile during the memory period. Consistent with our previous report^14^, the two neurons (red and blue) with RFs closer to the memorized location shift their RF toward that location during memory. The third neuron (green), a bit farther from that location, does not change its RF profile. Consequently, these changes can result in either an increase (between red and blue neurons) or decrease (between e.g., green and blue neurons) in the RF overlap between neurons, depending on how they change their RF profile during memory.

Out of 56 partially overlapping neuron pairs with the memory target presented at an In-flank location, we could obtain a well-shaped RF contour during the memory period for both neurons in 48 pairs (51 neurons). As expected, the RFs of these neurons shifted toward the remembered location (Fig. 5b bottom; n = 51 neurons, ∆RF distance_memory-fixation_ = −0.414 ± 0.168 d.v.a, *p* = 0.006, mean ± s.e.m) and expanded (Fig. 5b top; *n* = 51 neurons, ∆RF size_memory-fixation_ = 0.459 ± 0.229 d.v.a, *p* = 0.014, mean ± s.e.m). Figure 5c shows the shared RF ratio of 48 pairs of neurons during fixation and memory, where partially overlapping pairs are defined as those with shared RF ratios between 0.1 and 0.4. The 31 pairs of neurons for which the partially overlapping definition is met during fixation and memory are shown in black in Fig. 5c&e. Overall, these shifts and expansions resulted in some changes in RF overlap, although without any specific direction over the population (*n* = 48 pairs, ∆shared RF ratio_Inflank-fixation_ = −0.003 ± 0.021, *p* = 0.766, mean ± s.e.m; Fig. 5d, bottom histogram). In spite of the lack of a systematic change in RF overlap, we still looked for a relationship between the change in shared RF ratio and the effect of memory on the noise correlation. The top panel in Fig. 5d shows the noise correlation for memory IN (red) and OUT (blue), as a function of the change in shared RF ratio. During the memory IN condition, noise correlations are actually higher for pairs which have decreased their RF overlap—the opposite of what one would expect if changes in RF overlap were driving the noise correlations (Pearson correlation; *r* = −0.49, *p* = 0.0003). The increase in noise correlation between the memory IN and OUT conditions is independent of the change in RF overlap (Pearson correlation; *r* = −0.23, *p* = 0.116). These results indicate that the changes in RF profiles do not induce a systematic change in the RF overlap in the current dataset; furthermore, in general an increase in RF overlap during memory corresponded with a decrease in the correlated activity, similar to effects of sharing a top–down signal (Fig. 4) and opposite to the effects of sharing a bottom-up signal (Supplementary Figure 1).

We also performed a simpler control by using an even stricter criteria for identifying partially overlapping pairs and In-flank locations, limiting our analysis to pairs and locations which fit the definitions during both fixation and memory. For all 48 partially overlapping pairs, the noise correlation during In-flank memory is significantly higher than when remembering a location in opposite hemifield (Fig. 5e, all points; *n* = 48 pairs, ∆correlation_In-flank_ = 0.068 ± 0.019, *p* = 0.012). For the 31 pairs of neurons meeting the partially overlapping definition during both fixation and memory, there was still an increase in noise correlations when remembering the In-flank location compared to the location in opposite hemifield (Fig. 5e, black; *n* = 31 pairs; ∆Correlation_In-flank_ = 0.08 ± 0.029, *p* = 0.041). Thus although RF changes do occur during the memory period, the increase in noise correlation for partially overlapping pairs remains significant even when excluding pairs whose overlap category or sensitivity to the memory location has changed.

### Effects of visual and WM inputs on noise correlations

**Fig. 6:**
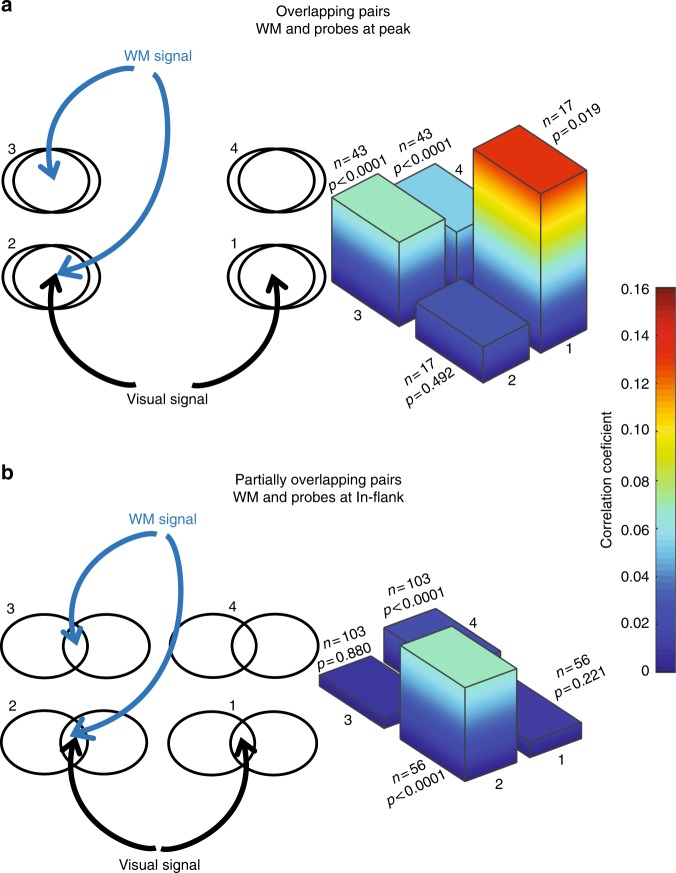
Noise correlations in the presence and absence of WM and visual signals. **a** Summary of noise correlations for overlapping pairs. The schematic on the left illustrates the conditions under which noise correlations were measured, with different combinations of top–down (WM) and bottom–up (visual) input in the Peak location, for overlapping pairs of neurons. The plot on the right shows the mean correlation coefficient for the populations of overlapping pairs, for all combinations of WM and visual input. Numbers (1–4) indicate which condition schematics on the left correspond to the noise correlation data on the right. WM signal (blue) is present when remembering a location in the RF, absent when remembering a location in the opposite hemifield. The visual signal (black) indicates data recorded on an MGS task with visual probes; different pairs of neurons were recorded with and without visual signals. **b** Summary of noise correlations for partially overlapping pairs. The schematic on the left illustrates the conditions under which noise correlations were measured, with different combinations of top–down (WM) and bottom–up (visual) input in the In-flank location, for partially overlapping pairs of neurons. The plot on the right shows the mean correlation coefficient for the populations of partially overlapping pairs, for all combinations of WM and visual input

Figure 6 summarizes the observed changes of noise correlations in overlapping and partially overlapping neuron pairs in the presence and absence of visual and WM signals. In the absence of a visual signal, the average noise correlation between 43 pairs of neurons with overlapping RFs increased with the addition of a WM signal. In the presence of a visual signal, the addition of a WM signal instead decreased the average noise correlation between 17 pairs of neurons with overlapping RFs. An interactive effect of the visual and WM signals on noise correlations was also observed for pairs of neurons with partially overlapping RFs: a WM signal reduced the noise correlation in the absence of visual signal (*n* = 103 pairs), but increased the noise correlation in the presence of a visual signal (*n* = 56 pairs). These findings show that although either a visual or WM signal alone increases the correlations between overlapping neurons, in combination these signals exert a decorrelating influence. This combination of results sets constraints on circuit level models of WM modulation.

## Discussion

Previous studies of noise correlations in visual cortex have examined correlations in spontaneous activity^16, 21^, the effect of stimulus presence and properties^22–24^, or the modulation of visual responses by cognitive factors^9, 10, 25–27^, but never the effect of localized top–down feedback in the absence of a visual stimulus. We sought to determine whether a top–down signal carrying the content of WM to extrastriate visual cortex is inherently correlative or decorrelative. The answer to this question is important for a better understanding of the neural circuitry involved in the control of visual signals and visual attention. We tested the effect of a top–down signal in isolation using a WM task in which neurons in extrastriate visual cortex are known to receive spatially specific input from the FEF part of prefrontal cortex^14^. In the absence of visual input, a WM signal increased correlations between MT neurons with spatially overlapping RFs. In contrast, a top–down signal decreased correlations between neurons with only partially overlapping RFs. The changes in noise correlations observed based on WM location and RF overlap in the absence of visual input (Figs. 2 & 3) are consistent with an excitatory, top–down input from PFC reflecting spatially specific WM activity of the sort which is known to exist in FEF^14, 28–30^ and dlPFC^31–34^ during this task. However, in the presence of a visual stimulus the direction of both these effects was reversed (Figs. 4 & 6). These findings now constrain models by which top–down signals modulate visual representations (Supplementary Note 8, Supplementary Figure 12).

The results indicate that neurons receiving the same top–down signal alone become more correlated. Recent findings from our lab suggest a mechanism within visual cortex that can serve as a common source of modulation. WM causes a spatially specific increase in the power of αβ oscillations in visual areas^15^. The degree to which neurons’ spike times lock to these oscillations also depends on the overlap between the neurons’ RF and the WM location^15^. Such changes in spike timing relative to a common oscillatory signal, when measured at an appropriate time scale across trials, affects correlated activity. Interestingly, in the presence of visual information, the spike-phase locking was altered based on the properties of the visual stimulus, potentially reducing the correlated activity. Changes in oscillatory properties within the visual area or in the top–down signal could both contribute to the observed changes in correlated activity. Untangling the exact network mechanism underlying these changes is beyond the scope of the current paper; however, one possibility is the activation of an inhibitory, decorrelating, network in the combined presence of top–down and bottom–up input. A simple schematic illustration of such a scenario is presented in Supplementary Figure 12.

The finding that the maintenance of a spatial memory is sufficient to decorrelate visual responses in extrastriate cortex is yet another similarity between the effects of spatial WM and covert attention (reviewed in Supplementary Discussion). Noise correlations can dramatically affect the information content of a neuronal population response^8, 11, 35–37^. These correlations are modulated by factors including attention^9, 10^, providing an additional means for attentional modulation to improve neuronal representations at the population level, and ultimately contribute to the perceptual benefits of attention. Indeed, changes in noise correlations have been suggested to be a key player underlying attention-induced representational enhancement^9^. By identifying a dissociation between the effects of a top–down signal in isolation and in the presence of visual input, these results provide a crucial step towards understanding the interactions between top–down and bottom–up signals and how this interaction alters correlated variability in sensory areas.

## Methods

### General & surgical procedures

Two adult male rhesus monkeys (*Macaca mulatta*) aged 5–7 years, were used in this study. All experimental procedures were in accordance with the National Institutes of Health Guide for the Care and Use of Laboratory Animals, the Society for Neuroscience Guidelines and Policies. The protocols for all experimental, surgical, and behavioral procedures were approved by the Montana State University Institutional Animal Care and Use Committee. The two animals used for these experiments were healthy in accordance with animal welfare standards as approved by the Montana State University Institutional Animal Care and Use Committee. All surgical procedures were carried out under Isoflourane anesthesia and strict aseptic conditions. Prior to undergoing behavioral training, each animal was implanted with a stainless steel headpost (Gray Matter Research, Bozeman, MT), attached to the skull using orthopedic titanium screws and dental acrylic. Following behavioral training, custom-made PEEK recording chambers (interior 22 × 22 mm) were mounted on the skull and affixed with dental acrylic. Within the chambers two 22 × 22 mm craniotomies were performed above the prefrontal and extrastriate visual areas (prefrontal chambers were centered at 42 mm A/P, 23 mm M/L and 28 mm A/P, and 23 mm M/L; extrastriate craniotomies were centered at −6 mm A/P, 23 mm M/L and −13 mm A/P, and 23 mm M/L).

### Behavioral monitoring

Animals were seated in a custom-made primate chair, with their head restrained and a tube to deliver juice rewards placed in their mouth. Eye position was monitored with an infrared optical eye tracking system (EyeLink 1000 Plus Eye Tracker, SR Research Ltd, Ottawa CA), with a resolution of <0.01º RMS; eye position was monitored and stored at 2 KHz. The EyeLink PM-910 Illuminator Module and EyeLink 1000 Plus Camera (SR Research Ltd, Ottawa, CA) were mounted above the monkey’s head, and captured eye movements via an angled infrared mirror. Juice was delivered via a syringe pump and the Syringe PumpPro software (NE-450 1L- X2, New Era Pump Systems, Inc., Farmingdale, NY). Stimulus presentation and juice delivery were controlled using custom software, written in MATLAB using the MonkeyLogic toolbox^38^. Visual stimuli were presented on an LED-lit monitor (Asus VG248QE: 24in, resolution 1920x1080, 144 Hz refresh rate), positioned 28.5 cm in front of the animal’s eyes. A photodiode (OSRAM Opto Semiconductors, Sunnyvale, CA) was used to record the actual time of stimulus appearance on the monitor, with a continuous signal sampled and stored at 32 KHz.

### Behavioral tasks

The fixation point, a ~1 dva white circle, appeared in the center of the screen, and the monkey maintained fixation within a ± 1.5 dva window for 1.5 s. For eye calibration the fixation point could appear either centrally or offset by 10 dva in the vertical or horizontal axis. The monkey was rewarded for maintaining fixation.

Preliminary RF mapping was conducted by having the monkey fixate within a ± 1.5 dva window around the central fixation point, while ~2.5 × 4 dva white bars swept in 8 directions (4 orientations) across the approximate location of the neuron’s RF. Responses from the recording site were monitored audibly and visually by the experimenter, and the approximate boundaries of the RF were noted for the positioning of stimuli in subsequent behavioral tasks.

RF mapping occurred during a passive fixation task. Monkeys fixated within a ± 1.5 dva window around the central fixation point. After 200 ms of fixation, RFs of neurons were mapped by presenting brief (200 ms) visual probes (~1 dva white circles) in a 7 × 7 dva grid of locations in 1–2.5 dva intervals. Eight probes were presented in succession, in pseudo-random order, with an inter-probe interval of 200 ms. This 7 × 7 grid of probes was positioned to overlap with the RF of the recorded neuron based on the preliminary RF mapping described above. Monkeys maintained fixation for 1800 ms to receive a reward.

Motion direction selectivity was measured during a visually guided saccade task. Monkeys fixated within a ± 1.5 dva window around the central fixation point. After 500 ms of fixation, a moving grating appeared in the RF of the neuron being recorded. After 1000 ms, the fixation point disappeared. On 50% of trials, a saccade target (1 dva black circle) appeared opposite the RF simultaneous with the offset of the fixation point; on these saccade away trials, the monkey saccaded away from the RF to the target to receive a reward. On the remaining 50% of saccade toward trials, no target appeared and the monkey saccaded to the moving grating to receive a reward. Gratings were 2.5 dva, 100% contrast, 1.5 degree/cycle and moved in one of eight directions.

During the memory guided saccade task, monkeys fixated within a ± 1.5 dva window around the central fixation point. After 1 s of fixation, a 1.35 dva circle target was presented and remained onscreen for 1 s. The animal then remembered the target location while maintaining fixation for 1 s (delay period) before the central fixation point was removed. The animal then had 500 ms to shift its gaze to a ± 4 dva window around the previous target location, and remain fixating there for 200 ms to receive a reward. This task was performed with six potential target locations, located in 45 degree increments from −90 to +90 degrees, and at 180 degrees relative to the estimated RF center.

During the memory guided saccade task with visual probes, monkeys fixated within a ± 1.5 dva window around the central fixation point. After 1 s of fixation, a 1.35 dva circular target was presented and remained onscreen for 1 s. The animal then remembered the target location while maintaining fixation for 1 s (delay period) before the central fixation point was removed. The animal then had 500 ms to move his eyes to a ± 4 dva window around the previous target location, and remain fixating there for 200 ms to receive a reward. RFs of neurons were mapped by presenting brief (200 ms) visual probes (~1 dva white circles) in a 7 × 7 dva grid of locations in 1–2.5 dva intervals, both before target presentation (baseline RF mapping) and during the delay period (delay period RF mapping). Four probes were presented in succession, with an inter-probe interval of 200 ms. This 7 × 7 grid of probes was positioned to overlap with the RF of the recorded neuron based on the preliminary RF mapping described above. The first probe from each trial was excluded from the analysis, to prevent contamination by a response to the offset of the memory target. The location of the remembered target could vary with respect to the RF of recorded neurons.

### Neurophysiological recording

The electrode was mounted on the recording chamber and positioned within the craniotomy area using a Narishige two-axis platform allowing continuous adjustment of the electrode position. For single-electrode recordings, a 28-gauge guide tube was lowered to contact or just penetrate the dura, using a manual oil hydraulic micromanipulator (Narishige, Tokyo, Japan). Then a varnish-coated tungsten microelectrode (FHC, Bowdoinham, ME), shank diameter 200–250 µm, impedance 0.2–1 MΩ (measured at 1 kHz), was advanced into the brain for the extracellular recording of neuronal activity. Single-electrode recordings used a Plexon pre-amplifier and AM Systems amplifier, filtering from 300 Hz-5 KHz. For array electrode recordings a 28-gauge guide tube was lowered as described, and the 16-channel linear array electrode (V-probe, Plexon, Inc., Dallas, TX) was advanced into the brain using the hydraulic microdrive. The array electrode was connected to a headstage pre-amplifier (Neuralynx, Inc., Bozeman, MT). Neuralynx Digital Lynx SX and associated software were used for data acquisition. Spike waveforms and continuous data were digitized and stored at 32 kHz for offline spike sorting and data analysis. Spike waveforms were sorted manually, and the quality of isolations for simultaneously recorded neurons confirmed using a support vector machine classifier (Supplementary Figure 13). Area MT was identified based on stereotaxic location, position relative to nearby sulci, patterns of gray and white matter, and response properties of units encountered. The location of brain areas within the recording chamber was verified via single-electrode exploration prior to beginning data collection with the electrode arrays.

### Data analysis

Units without visual responses or defined RFs were excluded. Statistical comparisons were performed using the Wilcoxon signed-rank test, except where otherwise noted. Sample sizes were not estimated by any statistical methods but were similar to what is generally accepted in the field. No randomization was used in the analysis. No blindness was used in the data collection and analysis.

Neuronal responses to the probes were measured in the window 30–230 ms after probe onset. For each neuron, the 7 × 7 probe responses were linearly interpolated to a 769 × 769 grid, and an RF profile was constructed based on locations in which probe-evoked activity was at least 50% of the maximum probe-evoked response. Shared RF area was the area of overlap between the two RF profiles.

1 $${\mathrm{Shared}}\,{\mathrm{RF}}\,{\mathrm{ratio}} = \sqrt {R1^\ast R2}$$

$${\mathrm{where}}\,{\mathrm{R1}} = \frac{{\rm{shared}}\,{\rm{RF}}\,{\rm{area}}}{{\rm{neuron1}}\,{\rm{RF}}\,{\rm{area}}}\,{\rm{and}\,{\rm{R2}}} = \frac{{{\rm{shared}\,{\rm{RF}\,{\rm{area}}}}}}{{{\rm{neuron2}\,{\rm{RF}\,{\rm{area}}}}}}$$

In Figs 2–6, pairs of neurons were sorted into overlapping and partially overlapping groups based on whether the shared RF ratio was greater than 0.5 or greater than 0.1 and less than 0.4, respectively.

Responses to eight directions of visual motion were recorded during the visually guided saccade task shown above. We first calculated the average visual response of each neuron to each of the eight directions of motion in the 30–1030 ms after stimulus onset. The similarity of feature tuning between two neurons was quantified as the correlation coefficient for the average firing rate of the two neurons in response to the eight motion directions.

Target and probe positions were described relative to the RF profiles of neuron pairs based on their responses at those locations during the RF mapping task. For pairs of neurons with overlapping RFs, peak locations were locations with responses >70% of maximum, Flanks were locations corresponding to 30–70% of the maximum response, and Tails were <30% of the maximum. For pairs of neurons with partially overlapping RFs, Peaks were locations with >70% for one neuron but <30% for the other, In-flank were locations with 30–70% of the maximal response for both neurons, and Out-flanks were locations with <30% of the maximal response for both neurons. For each pair we defined a location outside of both RFs if the geometric mean of both RF evoked responses was <30% of the maximum responses. If a memory target was presented at a location outside the area which was mapped during the RF mapping task, we used the location of adjacent memory targets to estimate the relative location of that WM target.

Noise correlations were calculated during the fixation period (Supplementary Figures 1&2), the delay period of the MGS task (Figs. 2&3), and in response to visual probes appearing during the delay period (Figs. 4&5). In the case of visual probes, correlation coefficients were calculated based on the cross-trial variability in the visual response to a single probe presentation (30–230 ms following onset of a probe at the same location for all trials). In each case the noise correlation was calculated over a 100 ms sliding window and then averaged across the period (200–1000 ms after fixation, 400–1000 ms after MGS target offset, and 30–230 ms after visual probe onset, respectively), using the Pearson correlation coefficient across trials. Across sessions, the mean number of trials used for calculating a correlation coefficient was *n* = 104 trials. When calculating the correlation values, we performed a rate-matching control using the method reported by^11^. In outline, this method involves looking at the firing rate for each neuron and time bin, and discarding points until a common firing rate distribution between conditions is achieved. To evaluate the significance of individual sample pairs’ noise correlations (Figs. 2&5), we obtained a null distribution by shuffling the trial pairings between the neurons 100 times, and calculating the noise correlation for each shuffled version; we then tested whether the actual noise correlation was significantly different from the mean of the shuffled distribution (Wilcoxon signed-rank test).

## Supplementary information

Supplementary Information

Peer Review

## Data Availability

Data and code used for analysis are available upon reasonable request.
