# Supplementary material for: Concurrent influence of top-down and bottom-up inputs on correlated activity of Macaque extrastriate neurons
Source: Nat Commun. 2018 Dec 19;9:5393. doi: 10.1038/s41467-018-07816-4 (PMC6300596; doi:10.1038/s41467-018-07816-4)
Supplement: Supplementary file 1 — Supplementary Information [file 41467_2018_7816_MOESM1_ESM.pdf]

**Merrikhi et al. "Concurrent influence of top-down and bottom-up inputs on correlated activity of Macaque extrastriate neurons"**

## Supplementary Information

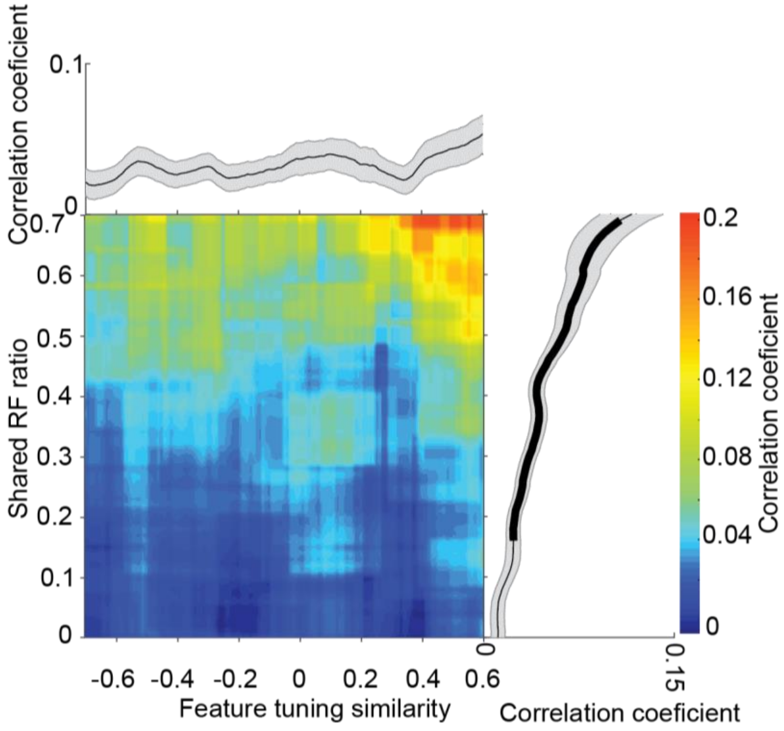


**Supplementary Figure 1**

Baseline noise correlations between MT neurons depend upon similarity of spatial RF but not feature tuning. The heatmap shows the mean noise correlation for pairs of neurons according to their similarity in feature tuning (x-axis) and RF overlap (y-axis); baseline noise correlation was measured during fixation with no visual stimulus in the neurons' RFs. There was no correlation between feature similarity and baseline noise correlation (r=0.008, p=0.663, linear regression). There was a correlation between RF overlap and baseline noise correlation (r=0.120, $p=9\times10^{-9}$, linear regression), but no significant interaction (r=0.037, p=0.394, linear regression). The plot to the right shows the noise correlation for the population as a function of RF similarity; the plot above shows the noise correlation for the population as a function of feature tuning similarity (marginal plots show mean baseline noise correlation value in a 0.3 sliding bin on the respective axis; bold portions indicate bins with mean noise correlation significantly different than zero, $p<10^{-4}$ Wilcoxon signed-rank test).

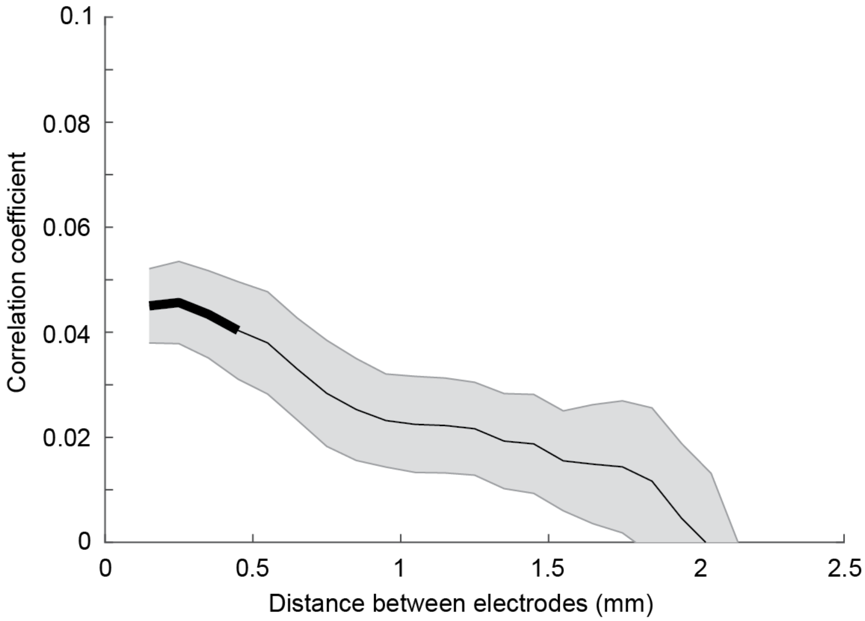


**Supplementary Figure 2**

Baseline noise correlations as a function of physical distance between neurons. Noise correlations between pairs of neurons during fixation, with no visual stimulus, are plotted as a function of the distance between the electrodes on which the neurons were recorded. As the distance between electrodes increases, the correlated variability of neurons decreases (Pearson correlation, $r=-0.15$, $p=0.005$). Bold portions indicate bins with mean noise correlation significantly different than zero, $p<10^{-3}$ Wilcoxon signed-rank test); distance is binned in a sliding window of 0.4 mm.

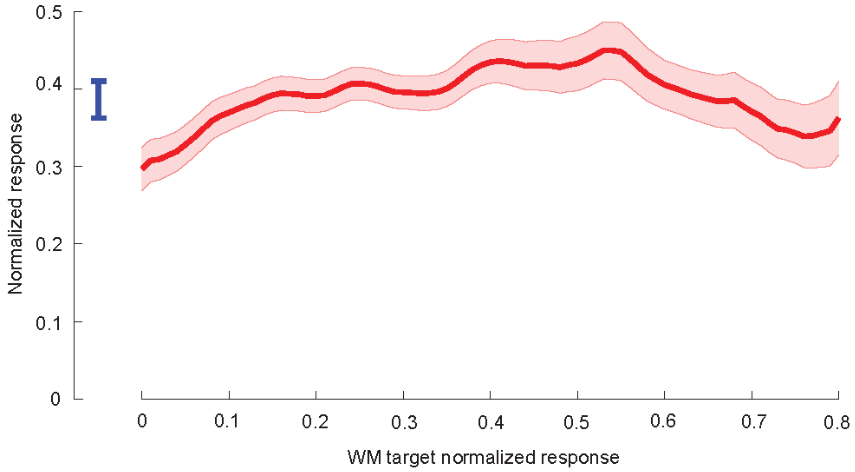


**Supplementary Figure 3**

The normalized delay period response of MT single neurons during different memory conditions. The response of each neuron is normalized (divided) by its own maximum response during the first 200 ms of the visual period across all conditions. The x-axis indicates the memory location relative to the location evoking the maximum visual response for each neuron, and is binned in units of 0.2. The red graph shows the normalized delay period response of 85 single MT neurons during the memory IN condition; the blue error bar shows the mean normalized delay period response for the same population during the memory OUT condition (mean ± s.e.m). There is no significant dependence of the delay period response of MT single neurons on which of the five memory IN locations was held in memory (r=0.082, p=0.167). There is also no significant change in the delay period firing rate based on whether the location held in WM was in the same or the opposite hemifield from the neuron's RF (n=85, $\Delta FR_{IN-OUT}$=-0.22 ± 0.15 Hz, p=0.656).

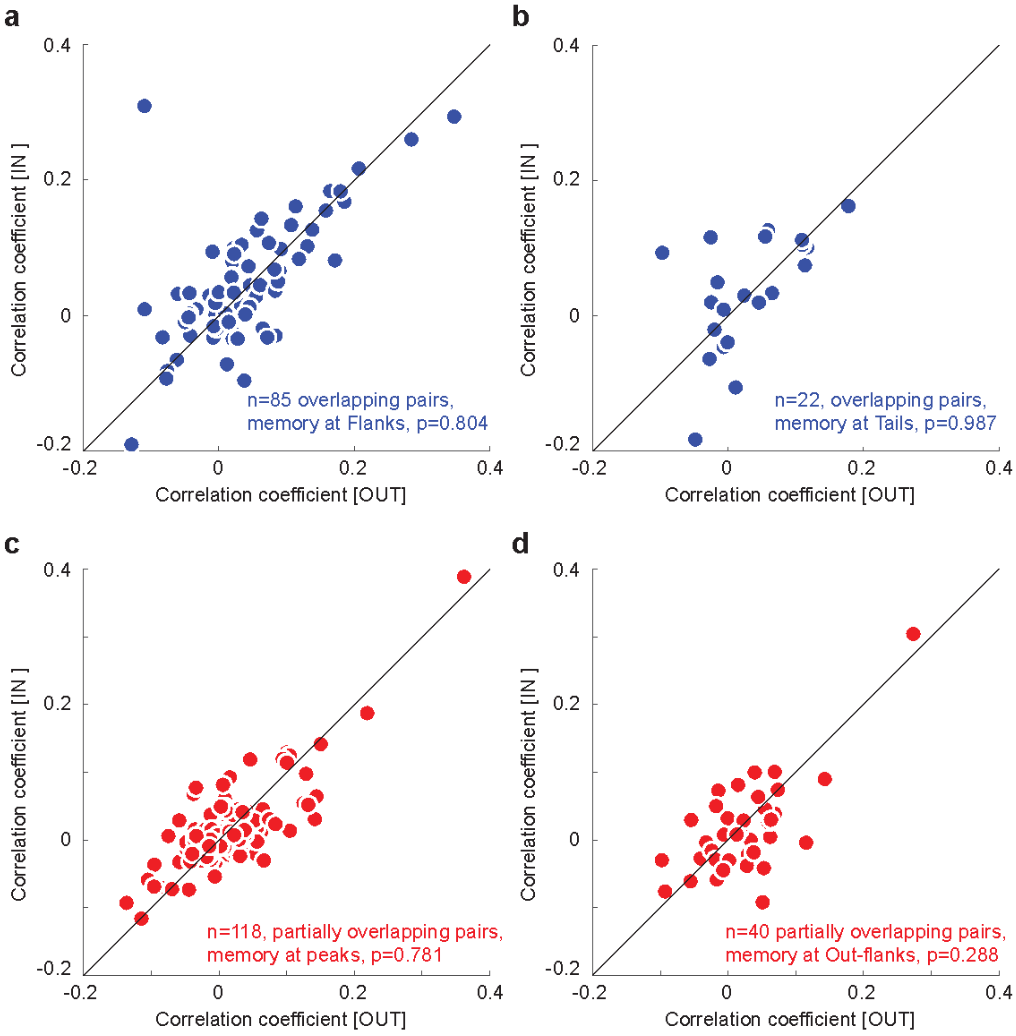


**Supplementary Figure 4**

Noise correlations are unchanged for other combinations of RF overlap and memory location, in the MGS task with no visual probes. All plots compare noise correlations for pairs of neurons during memory OUT (x-axis) and memory IN (y-axis). (a) No change in noise correlations based on memory location for overlapping pairs with memory in a Flanks location (n=85 pairs, p=0.804). (b) No change in noise correlations based on memory location for overlapping pairs with memory in a Tails location (n=22 pairs, p=0.987). (c) No change in noise correlations based on memory location for partially overlapping pairs with memory in a Peak location (n=118 pairs, p=0.781). (d) No change in noise correlations based on memory location for partially overlapping pairs with memory in an Out-flanks location (n=40 pairs, p=0.288).

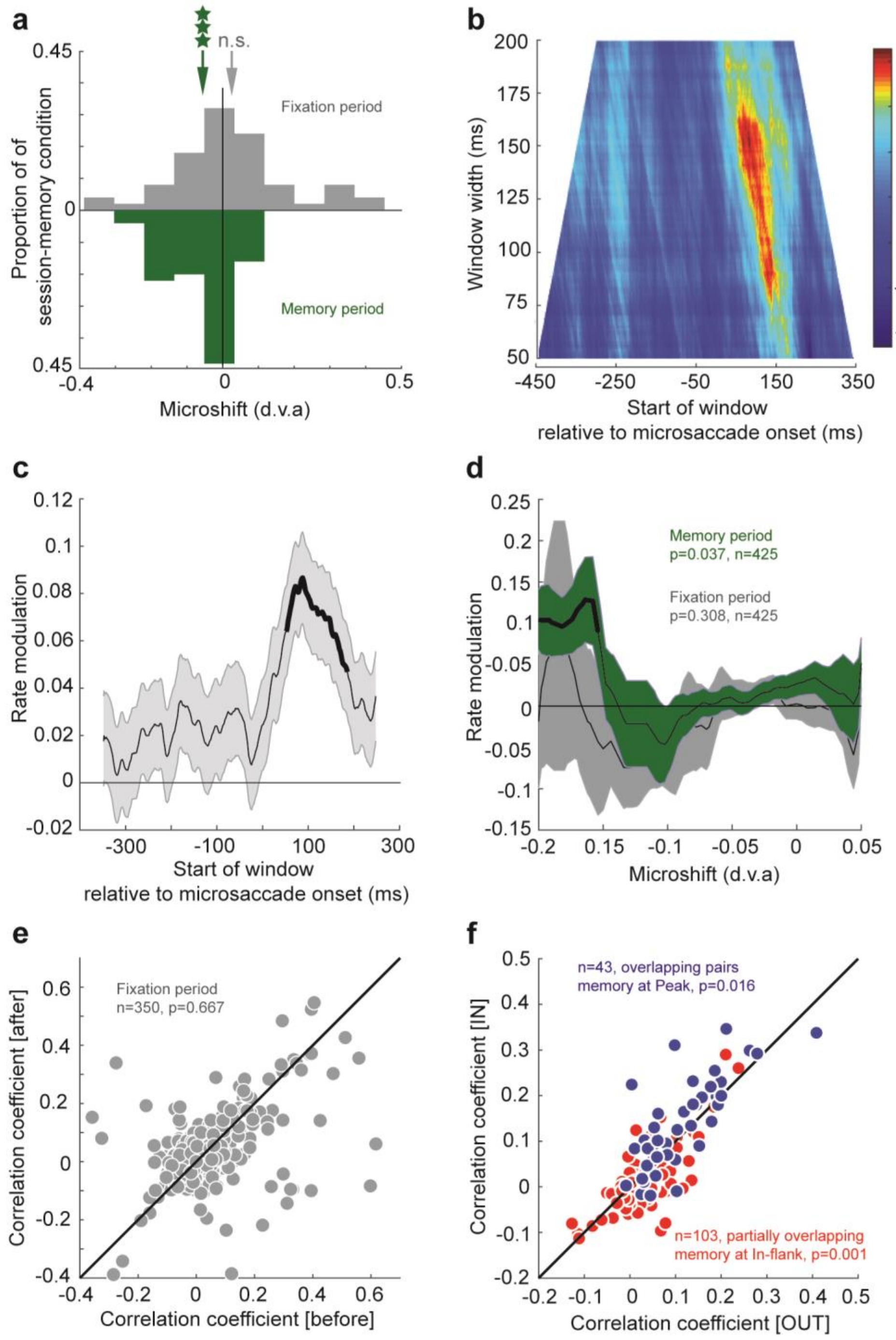


**Supplementary Figure 5**

Microsaccades do not account for changes in noise correlations. (a) During fixation, microsaccades are not biased relative to the future memory location. Gray histogram shows the distribution of microshifts for each session and memory location; there is no significant microshift (n=55 session-location combinations, p=0.348). Negative microshifts indicate microsaccades toward the memory location. During memory, microsaccades were biased toward the memory location. Green histogram shows the distribution of microshifts during the memory period; a

negative mean indicates that microsaccades were more often directed toward the memory location (n=55 session-location combinations, p=0.0004) (b) A rate modulation index was calculated using sliding windows with different widths starting at different times relative to the onset of microsaccades. The maximum rate modulation was observed for a window width of 150 ms starting 90 ms after the microsaccade. (c) The rate modulation index measured with a 150 ms sliding window at different times relative to microsaccade onset. The bold portion shows times with significant rate modulation after microsaccade onset (p<0.05). (d) During memory, firing rate increases follow microsaccades toward the memory location. Green plot shows firing rate modulation within 90-240 ms after each microsaccade compared to 150-0 ms before each microsaccade as a function of microsaccade direction, during the memory period. Gray plot shows the firing rate modulation during the fixation period. During the fixation period there is no overall change in firing rate of neurons after microsaccades and no relationship between the direction of microsaccades and rate modulation. (e) During fixation, there was no change in noise correlations following microsaccades (n=350, p=0.667). (f) Changes in noise correlations are still present when excluding the 90-240 ms after each microsaccade. Scatter plot shows a comparison of noise correlation values for memory IN vs. OUT, excluding data from 90-240 ms after a microsaccade. Noise correlations for overlapping pairs increased when remembering a Peak location (blue; n=43, p=0.016). Noise correlations for partially overlapping pairs decreased when remembering an In-flank location (red; n=103, p=0.001).

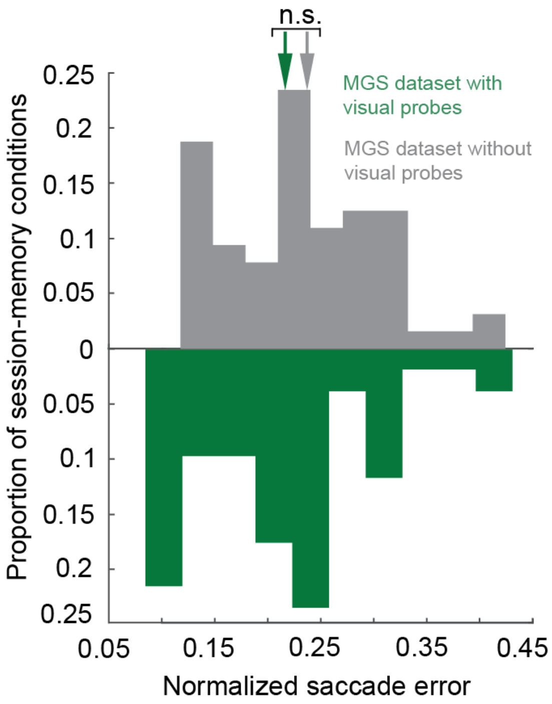


**Supplementary Figure 6**

Saccade error is not altered by the presence of visual probes. Histograms show the distribution of normalized saccade error on the MGS task across sessions and target locations, for sessions with (green) or without (gray) visual probes. There was no difference in average saccade error for sessions with vs. without visual probes ($p=0.106$).

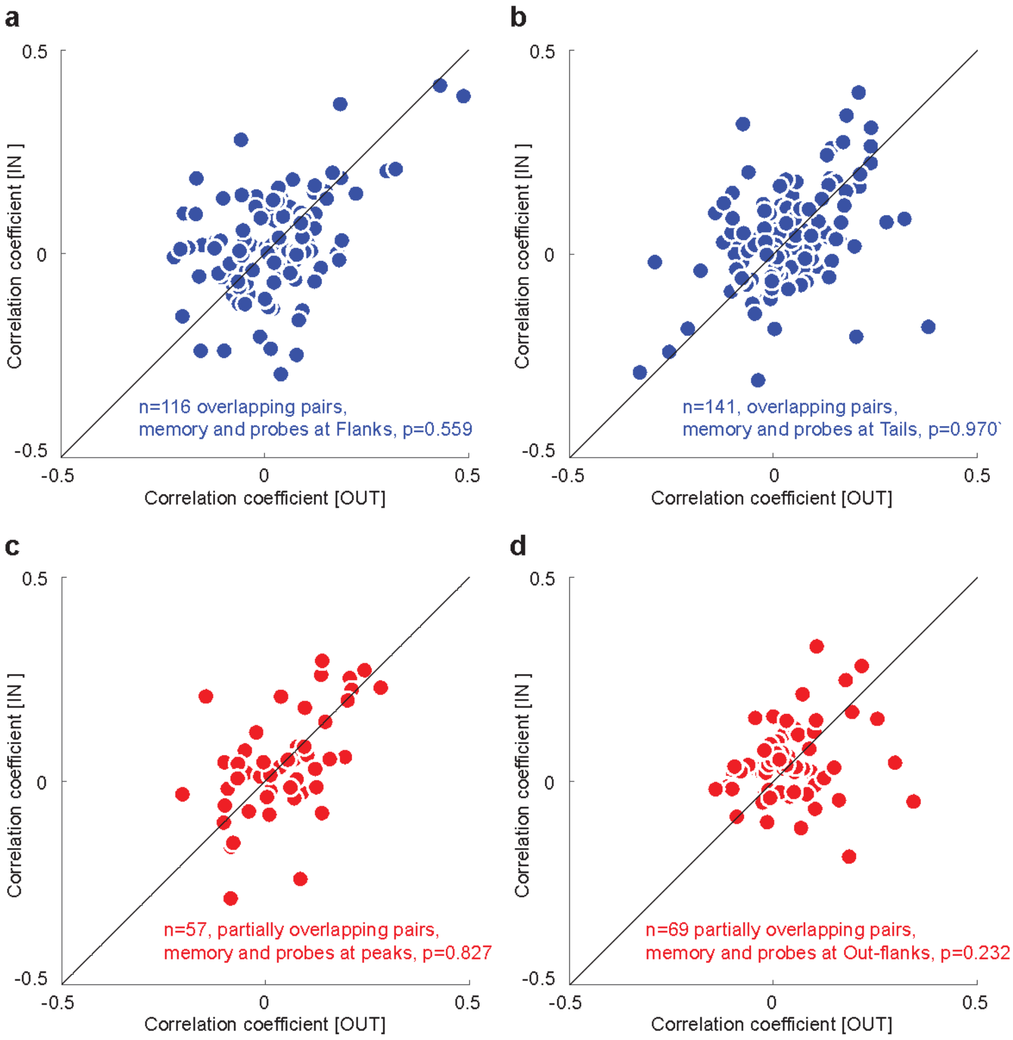


**Supplementary Figure 7**

Noise correlations are unchanged for other combinations of RF overlap and memory location, in the MGS task with visual probes. All plots compare noise correlations for pairs of neurons during memory OUT (x-axis) and memory IN (y-axis). (a) No change in noise correlations based on memory location for overlapping pairs with memory in a Flank location (n=116 pairs, p=0.559). (b) No change in noise correlations based on memory location for overlapping pairs with memory in a Tails location (n=141 pairs, p=0.970). (c) No change in noise correlations based on memory location for partially overlapping pairs with memory in a Peak location (n=57 pairs, p=0.827). (d) No change in noise correlations based on memory location for partially overlapping pairs with memory in an Out-flanks location (n=69 pairs, p=0.232).

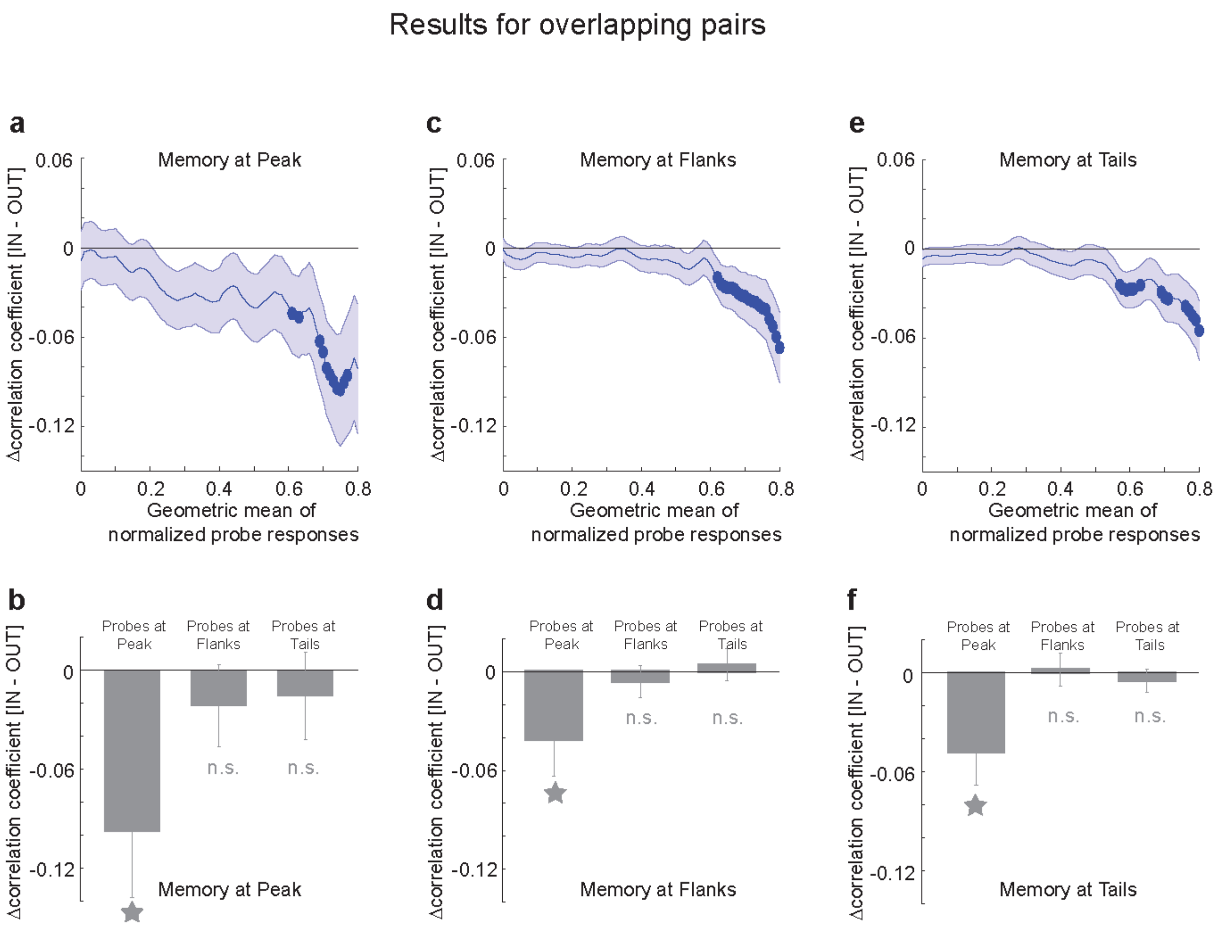


**Supplementary Figure 8**

Changes in noise correlations as a function of visual probe location, for overlapping neuron pairs. (**a**) Changes in noise correlations of overlapping pairs of neurons: remembering a target in the Peak location decreases the noise correlation for probes appearing at high response RF locations (corresponding to the Peak designation). Probe location (x-axis) here and in (**c** and **e**) is plotted according to the geometric mean of the two normalized RF profile responses at that location. The change in noise correlation is plotted for neuron pairs in a 0.3 wide bin across probe locations (x-axis); areas in bold had a significant change in noise correlation ($p<0.05$). (**b**) When remembering a Peak location, overlapping neuron pairs showed a significant decorrelation for visual responses to probes appearing in the Peak location ($\Delta correlation_{IN\text{-}OUT}$=-0.099 ± 0.040, p=0.014, n=59 pair-probes), but no effects for visual probes appearing in the Flanks or Tails (Flanks, $\Delta correlation_{IN\text{-}OUT}$=-0.022 ± 0.025, p=0.940, n=154 pair-probes; Tails, $\Delta correlation_{IN\text{-}OUT}$= -0.016 ± 0.027, p=0.675, n=162 pair-probes). (**c**) For overlapping pairs of neurons, remembering a target in the Flanks location does not significantly change the noise correlation for probes corresponding to the Flanks designation. (**d**) When remembering a Flanks location, overlapping neuron pairs showed a significant decorrelation for visual responses to probes appearing in the Peak location ($\Delta correlation_{IN\text{-}OUT}$=-0.041 ± 0.022, p=0.014, n=235 pair-probes), but no effects for visual probes appearing in the Flanks or Tails (Flanks, $\Delta correlation_{IN\text{-}}$

$_{OUT}$=-0.006 ± 0.010, p=0.692, n=1226 pair-probes; Tails, $\Delta$correlation$_{IN\text{-}OUT}$=-0.004 ± 0.010, p=0.444, n=1026 pair-probes). (**e**) For overlapping pairs of neurons, remembering a target in the Tails location does not significantly change the noise correlation for probes corresponding to the Tails designation. (**f**) When remembering a Tails location, overlapping neuron pairs showed a significant decorrelation for visual responses to probes appearing in the Peak location ($\Delta$correlation$_{IN\text{-}OUT}$=-0.048 ± 0.020, p=0.031, n=226 pair-probes), but no effects for visual probes appearing in the Flanks or Tails (Flanks, $\Delta$correlation$_{IN\text{-}OUT}$=0.002 ± 0.010, p=0.712, n=1029 pair-probes; Tails, $\Delta$correlation$_{IN\text{-}OUT}$=-0.005 ± 0.007, p=0.560, n=1969 pair-probes). Data are shown as mean ± s.e.m.

Results for partially overlapping pairs

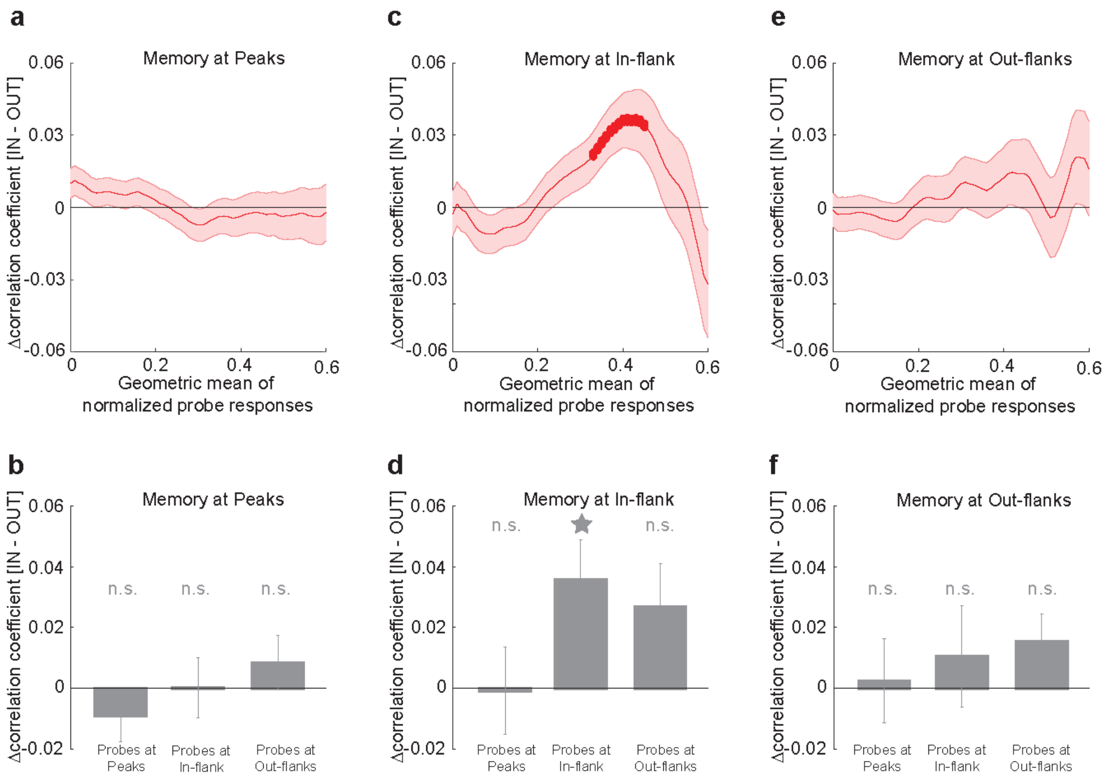


**Supplementary Figure 9**

Changes in noise correlations as a function of visual probe location, for partially-overlapping neuron pairs. (**a**) Changes in noise correlation of partially overlapping pairs of neurons: remembering a target in the Peaks location does not significantly alter the noise correlation for responses to probes corresponding to the Peaks designation. Probe location (x-axis) here and in (**c** and **e**) is plotted according to the geometric mean of the two normalized RF profile responses at that location. Change in noise correlation is plotted for neuron pairs in a 0.3 wide bin across probe locations (x-axis); areas in bold had a significant change in noise correlation ($p<0.05$). (**b**) When remembering a Peaks location, partially-overlapping neuron pairs showed no significant change for visual responses to probes appearing in the Peaks, In-flank or Out-flanks location (Peaks, $\Delta correlation_{IN-OUT}$=-0.009 ± 0.009, p=0.310, n=1400 pair-probes; In-flank, $\Delta correlation_{IN-OUT}$=0.001 ± 010, p=0.952, n=1037 pair-probes; Out-flanks, $\Delta correlation_{IN-OUT}$=0.008 ± 0.009, p=0.315, n=1144 pair-probes). (**c**) For partially-overlapping pairs of neurons, remembering a target in the In-flank location significantly increases the noise correlation for probes corresponding to the In-flank designation. (**d**) When remembering an In-flank location, partially-overlapping pairs showed a significant increase in correlation for probes appearing in the In-flank location (In-flank, $\Delta correlation_{IN-OUT}$=0.036 ± 0.013, p=0.031, n=535 pair-probes), but no change for probes in the Peaks or Out-flanks (Peaks, $\Delta correlation_{IN-OUT}$=-0.001 ± 0.014, p=0.854, n=438 pair-probes; Out-flanks, $\Delta correlation_{IN-OUT}$=0.027 ± 0.014, p=0.130, n=392 pair-probes). (**e**) For partially-overlapping pairs of neurons, remembering a target in the Out-flanks

location does not significantly alter the noise correlation for probes corresponding to the Out-flanks designation. (**f**) When remembering an Out-flanks location, partially-overlapping neuron pairs showed no significant change for visual responses to probes appearing in the Peaks, In-flank or Out-flanks locations (Peaks, $\Delta correlation_{IN-OUT}=0.003 \pm 0.014$, p=0.746, n=522 pair-probes; In-flank, $\Delta correlation_{IN-OUT}=0.010 \pm 0.017$, p=0.934, n=450 pair-probes; Out-flanks, $\Delta correlation_{IN-OUT}=0.015 \pm 0.009$, p=0.196, n=902 pair-probes). Data are shown as mean ± s.e.m.

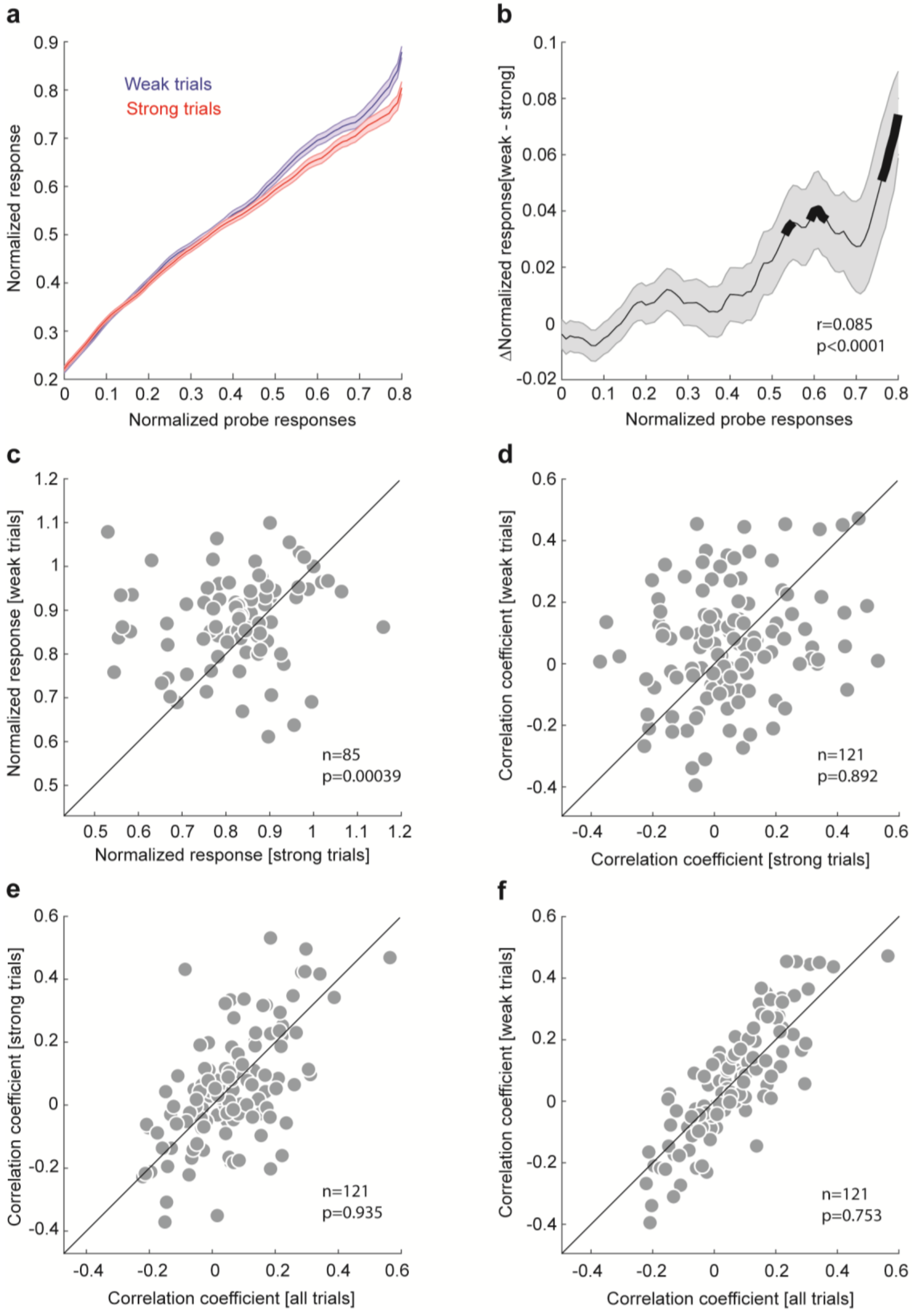


**Supplementary Figure 10**

Adaptation: recent firing rate history alters visual responses but not noise correlations. (a) Effects of firing rate history on visual responses, as a function of probe efficacy, for 114 MT neurons. Responses to probes at different locations are binned along the x-axis based on the fraction of

the neuron's maximum response evoked by the probe. The responses of each neuron to a single probe are split into strong (red) and weak (blue) trials, based on whether the response to the previous probe was higher (strong trial) or lower (weak trial) than the median of all that neuron's probe-evoked responses. Across all probe locations there was an adaptation effect: trials in which an effective stimulus preceded the probe had lower firing rates than those preceded by an ineffective stimulus (n=114 neurons, $\Delta$normalized FRweak-strong=0.012 ± 0.004, p=0.003). (b) Adaptation of responses is stronger for effective probes. Plot shows change in probe-evoked response based on recent history (weak-strong), as a function of probe efficacy. The strength of the change in firing rate (adaptation) was correlated with probe efficacy (r=0.085, p<0.0001). (c) Adaptation reduces responses to Peak probe positions for 85 MT neurons. Plot shows probe-evoked responses to probes appearing in a Peak location, for weak and strong trials. Responses of neurons were significantly reduced in strong trials (n=85 neurons, $\Delta$normalized FR weak-strong=0.051 ± 0.015, p=0.00039). The 85 neurons in this plot are the members of the overlapping pairs in (d). (d) Firing rate history does not alter noise correlations in 121 overlapping pairs of neurons. Plot shows noise correlation values of probe-evoked responses to probes appearing in the Peak of the neurons' RFs, for weak and strong trials. There was no significant change in noise correlations for weak vs. strong trials (n=121 pairs, $\Delta$correlation weak-strong=0.006 ± 0.017, p=0.892). (e) There was no significant change in noise correlations for strong vs. all trials ($\Delta \text{correlation}_{\text{strong-all}}$=-0.004 ± 0.011, p=0.935, n=121 pairs). (f) There was also no significant change of noise correlations for weak vs. all trials ($\Delta \text{correlation}_{\text{weak-all}}$=0.002 ± 0.008, p=0.753, n=121 pairs).

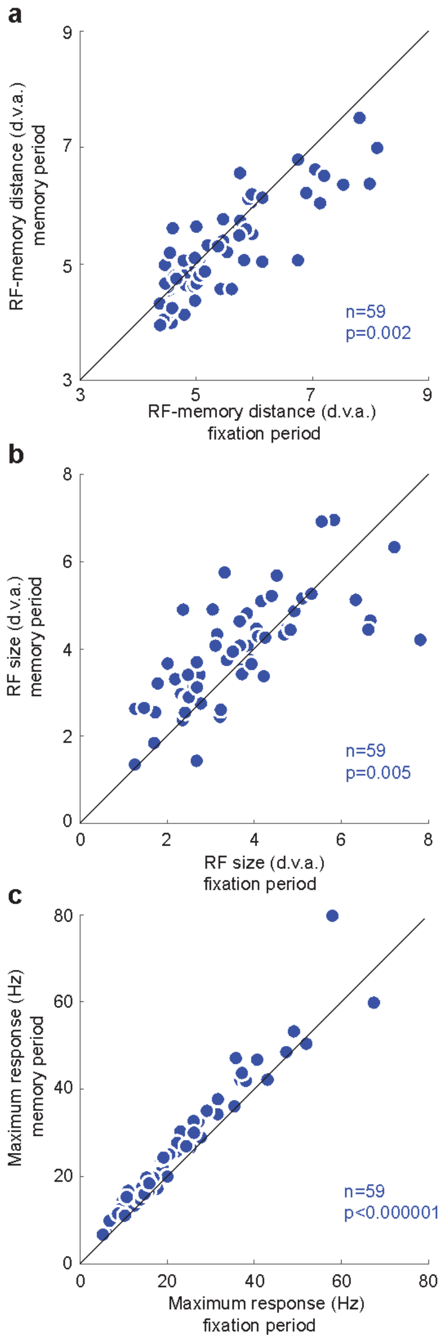


**Supplementary Figure 11**

Changes in RF position, size, and response gain during WM for 59 MT neurons. (a) RFs shift toward the memory location. Plot shows the average of distance between the RF center and the memory location during the three memory IN conditions (y-axis) and fixation (x-axis). During memory, the RF center shifts closer to the memory location (n=59 neurons, $\Delta distance_{memory-fixation}=-0.230 \pm 0.067$, p=0.002). (b) RFs expand during memory. Plot shows the average RF size during the memory IN conditions (y-axis) and fixation (x-axis). During memory, RF size increases ($\Delta RF\ size_{memory-fixation}=-0.288 \pm 0.131$, p=0.005). (c) Visual gain increases during memory. Plot shows the maximum response to a visual probe during fixation (x-axis) and the average of the maximum responses during the three memory IN conditions (y-axis). Peak responses increase during memory ($\Delta max\ response_{memory-fixation}=3.239 \pm 0.450$ Hz, $p<10^{-6}$).

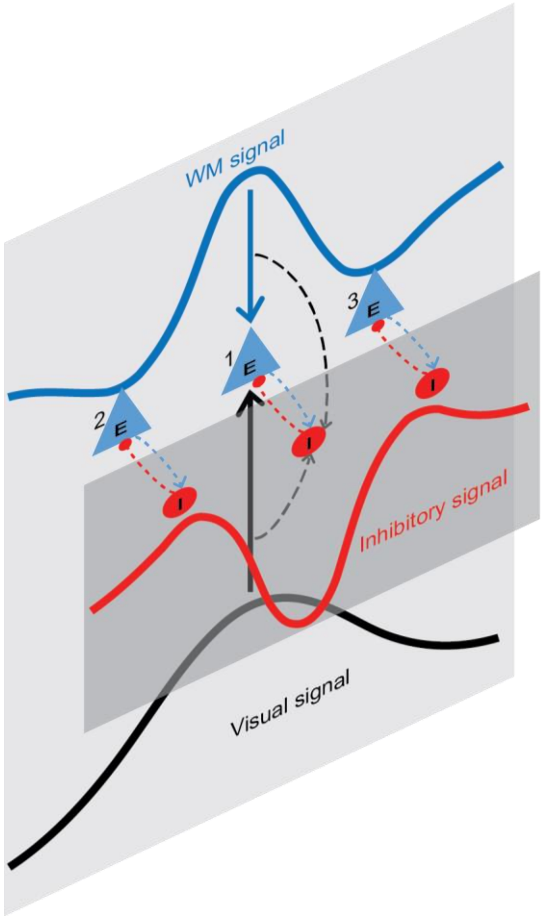


**Supplementary Figure 12**

A conceptual model accounting for the observed levels of noise correlations. Blue triangles indicate pools of excitatory neurons with overlapping RFs; excitatory pools 2 & 3 correspond to neurons whose RFs are partially-overlapping with excitatory pool 1. These pools of excitatory neurons receive excitatory visual (black arrow) and WM inputs (blue arrow); either of these signals in isolation increases correlations between neurons with overlapping RFs (i.e., in the same pool). Red circles indicate pools of inhibitory neurons reciprocally connected to an excitatory pool with overlapping RFs. These local inhibitory pools are only active (or only active enough to influence network correlations) when receiving both visual and WM signals. The WM, inhibitory, and visual profiles all indicate the similarity of signal from that source for a pair of neurons, one located in group 1, the second located at the horizontal location of the plot- similar signals cause correlations between neurons, dissimilar signals cause decorrelations. To fully account for the observed changes in noise correlations, there must also be a broader inhibitory circuit within either MT or FEF, not illustrated here. Grouping neurons into discrete pools representing neurons with overlapping vs. partially-overlapping RFs is for illustrative purposes only: changes in connectivity and shared input over cortical space are likely continuous rather than discrete.

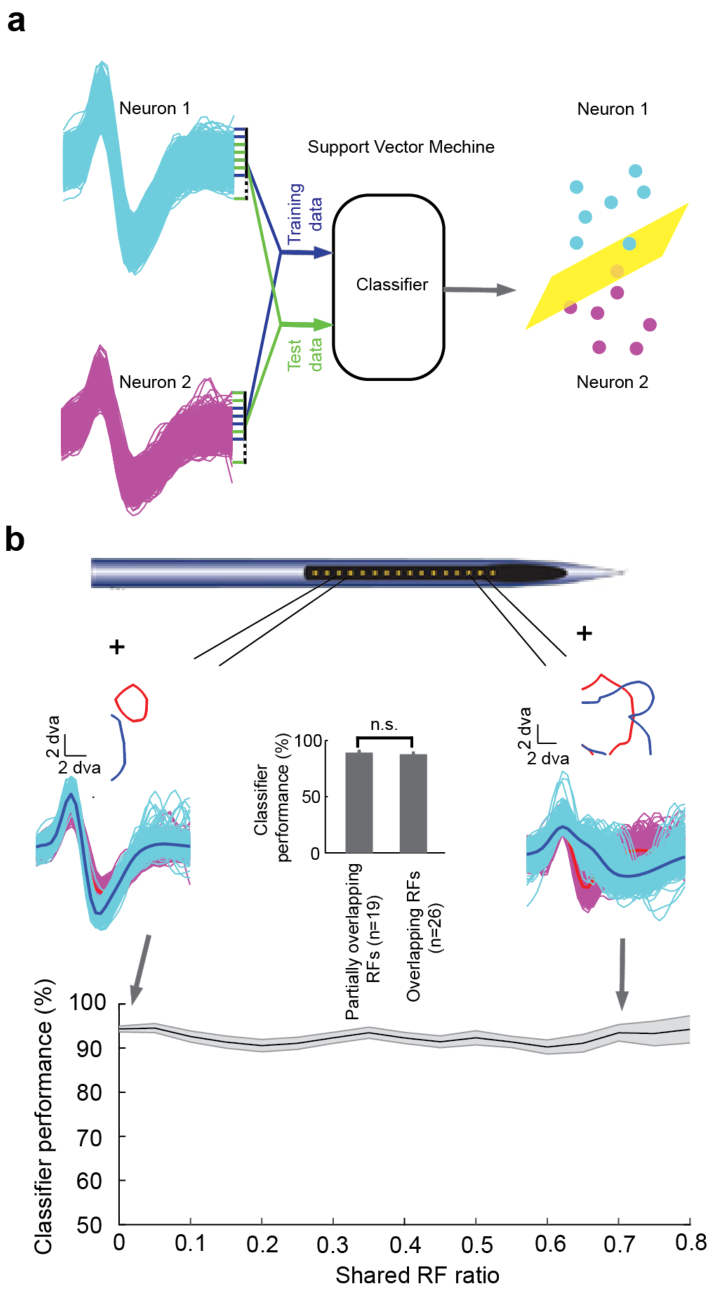


## Supplementary Figure 13

SVM control for the quality of neuronal isolations. (**a**) Schematic of the SVM classifier used to test the isolation quality of two spike waveforms recorded during the same session (n=350 pairs). In order to make sure that the manually sorted neurons are well isolated, we used Support Vector Machine (SVM) classifiers with linear kernels to categorize the spikes of simultaneously recorded pairs of neurons. To do that, we applied the SVM to a window of 200 spikes from each neuron in a pair, and slid that 200-spike window through the recording session. Within each window, we randomly selected 100 spikes from each neuron to train the classifier (training data, blue) and used the remaining 100 spikes to test the classifier performance (test data, green). The SVM classified each waveform as belonging to one of the two neurons, and classifier performance was compared to the assignment based on manual spike sorting. We averaged the

classifier performance across different windows over the session to quantify how well the spikes from each neuronal pair are isolated. (**b**) Classifier performance is high and independent of RF overlap between the neurons. The main plot shows the average classifier performance in categorizing the spike waveforms of simultaneously recorded neurons as a function of the overlap between the neurons' RFs. The plot was obtained using a 0.1 sliding bin on the x-axis. The overall high performance of the classifier indicates that the spikes of neuronal pairs are well isolated (the mean classifier performance across all the pairs=92% ± 0.46, n=350, $p=1\times10^{-20}$ compared to chance level, i.e. 50%). Classifier performance is high across a range of shared RF ratios, and there is no significant dependence of classifier performance on the overlap between neurons' RFs (Pearson correlation; correlation coefficient =-0.06, p=0.196, n=350). The RF contours and spike waveforms of two sample pairs, which were recorded on adjacent contacts of the array electrode, are shown. The RFs of the sample pair shown on the left (recorded by contacts #13 and #14) have no overlap, while the RFs of the sample pair shown in right (recorded by contacts #2 and #3) are highly overlapping (gray arrows indicate the average classifier performance at the RF overlap values of these pairs). For further analysis, we focused on all the neuronal pairs which were recorded on recording contacts in close proximity to one another (distance<200 µm; n=45 pairs), and grouped them into similar (shared RF ratio>0.5) and dissimilar RFs based on their RF overlaps (shared RF ratio<0.5). The bar plot shows the mean classifier performance in categorizing the spike waveforms of pairs of neurons with similar and dissimilar RFs. The result shows that there is no significant difference between the classifier performances for these two groups of neuronal pairs (the mean classifier performance for pairs with similar RFs =88% ± 2.45, n=26, the mean classifier performance for pairs with dissimilar RFs=89% ± 2.47, n=19, p=0.954, Wilcoxon rank-sum test). Data are shown as mean ± s.e.m.

## Supplementary Note 1

## Modulation of sensory areas during WM and attention

During visuospatial attention, activity in a broad network of frontal, parietal, and subcortical structures reflects the locus of attention, and a signal sent from this network to visual areas is believed to enhance the processing of signals within these areas, ultimately resulting in the behavioral benefits of attention[1,2]. A top-down signal from prefrontal cortex (PFC) is considered to be a likely source of attentional modulations in posterior visual cortex[1,3–5], and indeed a spatially-selective signal, reflecting the content of working memory (WM), sent directly from the Frontal Eye Field (FEF) to visual areas might be the means by which prefrontal cortex alters representations within sensory areas[6]. Importantly, although the firing rate of sensory areas seems to be unaltered by this signal in the absence of sensory stimuli, the local field potential (LFP) power spectra and spike timing reflect the WM content[7]. These results provide strong support for the theory of sensory recruitment during WM: sensory areas are recruited by WM to maintain a rich representation of information in order to guide behavior[8]. These experiments identified one source of the WM signal sent to visual areas, and demonstrated the subthreshold modulation of LFPs in these areas, which now motivates us to ask how a top-down spatial signal affects the inter-neuronal relationships of neurons in visual areas.

If neurons in visual areas are altered by a shared top-down signal, then the question is how such a common input decorrelates activity within visual areas, as is observed during covert attention[9–11]. Some possible scenarios are: 1) The shared top-down signal is inhibitory (either the projections themselves are inhibitory, or excitatory input targets inhibitory neurons within visual areas). This scenario is unlikely considering that projections from the FEF to visual areas are excitatory and target primarily pyramidal neurons rather than interneurons within visual areas [12]. Moreover, in causal experiments an increase or decrease in the responses of visual neurons always follows the activation or inactivation of the FEF, respectively. 2) The effect may not always be decorrelative, but instead depend on the properties of the neurons and the modulatory signal. Attention studies have looked at neurons receiving diverse top-down signals, and reported both correlation and decorrelation effects[19]. The degree to which these effects depend on spatial receptive field overlap and feature tuning similarity between pairs of neurons has yet to be precisely measured. 3) A common top-down signal can recruit different networks in sensory areas resulting in an overall reduction of correlated activity.

## Supplementary Note 2

## RF overlap, not physical distance between neurons, determines noise correlation

It is important to establish whether the relationship between noise correlation and RF overlap is truly dependent on the RF similarity, or instead a secondary consequence of the relationship between RF overlap and cortical distance. As expected given the retinotopic organization of MT cortex, there is a correlation between cortical distance (measured as the

distance between electrode contacts on which neurons were recorded) and RF overlap values for neuron pairs (r=-0.378, $p<10^{-12}$). Accordingly, there is an inverse correlation between the noise correlation and the physical distance between the contacts where neurons were recorded: as the distance between electrodes increases the correlated variability of neurons decreases (Pearson correlation, r=-0.15 p=0.005; Supplementary Fig. 2). Importantly, however, a multi-variable linear regression factoring in both physical distance and RF overlap indicates that RF overlap is the crucial factor in predicting noise correlations: the noise correlation between neurons is dependent on the degree of overlap between their spatial RFs (r=0.140, $p<10^{-3}$, linear regression), but not the distance between electrodes ($r=4\times10^{-6}$, p=0.751, linear regression; no significant interaction between spatial tuning and distance: $r=-2\times10^{-5}$, p=0.441, linear regression). Thus RF overlap, rather than feature tuning or physical distance, is the best predictor of noise correlations during fixation.

## Supplementary Note 3

### No changes in noise correlations for other memory locations, with or without visual probes

For the pairs with overlapping RFs, remembering a location in the Flanks or Tails of the RFs had no significant effect on the noise correlation (Supplementary Fig. 4a&b, n=85 pairs, $\Delta correlation_{Flanks}$=0.002 ± 0.007, p=0.804; n=22 pairs, $\Delta correlation_{Tails}$=0.005 ± 0.015, p=0.987). For the pairs with partially-overlapping RFs, remembering a location in the peak of one RF or an Out-flanks location had no effect on the correlation between neurons (Supplementary Fig. 4c&d, n=118 pairs, $\Delta correlation_{Peaks}$=-0.0001 ± 0.004, p=0.781; n=40 pairs, $\Delta correlation_{Out\text{-}flanks}$=-0.010 ± 0.008, p=0.288). In the presence of visual probes, no significant changes in the noise correlations were observed for memory locations in the Flanks or Tails locations for overlapping pairs (Supplementary Fig. 7a&b, n=116 pairs, $\Delta correlation_{Flanks}$=-0.003 ± 0.012, p=0.559; n=141 pairs, $\Delta correlation_{Tails}$=0.001 ± 0.015, p=0.970). For partially-overlapping pairs, no significant change was observed in the Peaks or Out-flanks conditions (Supplementary Fig. 7c&d, n=57 pairs, $\Delta correlation_{Peaks}$=0.008 ± 0.019, p=0.827; n=69 pairs, $\Delta correlation_{Out\text{-}flanks}$=0.004 ± 0.014, p=0.233).

## Supplementary Note 4

### No changes in noise correlations for non-overlapping pairs, with or without visual probes

Non-overlapping pairs (RF ratio <0.001) showed no significant changes in noise correlation in the absence of visual probes, for any memory location ($\Delta correlation_{Peaks}$=0.016 ± 0.014, p=0.5, n=2 pairs; $\Delta correlation_{In\text{-}flank}$=-0.018 ± 0.015, p=0.301, n=9 pairs; $\Delta correlation_{Out\text{-}flanks}$=-0.001 ± 0.009, p=0.952, n=14 pairs). In the presence of visual probes, non-overlapping

pairs also showed no significant changes in noise correlation for memory in the Peaks location, in the presence of visual probes at any location ($\Delta correlation_{Peaks}$=0.118 ± 0.061, p=0.151, n=32 pair-probe; $\Delta correlation_{In\text{-}flank}$=0.095 ± 0.099, p=0.391, n=20 pair-probe; $\Delta correlation_{Out\text{-}flanks}$=0.027 ± 0.047, p=0.757, n=37 pair-probe), nor for memory in the Out-flank location, in the presence of visual probes at any location ($\Delta correlation_{Peaks}$=0.016 ± 0.039, p=0.280, n=70 pair-probe; $correlation_{In\text{-}flank}$=-0.089 ± 0.052, p=0.103, n=41 pair-probe; $\Delta correlation_{Out\text{-}flanks}$=0.001 ± 0.022, p=0.901, n=235 pair-probe). (There were not any non-overlapping pairs with the memory location at the In-flank location in the visual probe dataset.)

## Supplementary Note 5

### Microsaccades do not account for the changes in noise correlations during WM

In light of recent results emphasizing the importance of microsaccades during covert attentional modulations of extrastriate visual cortex[13–16], we investigated what role, if any, microsaccades played in driving the changes in noise correlations during WM. For each microsaccade we calculated the microshift, defined as the difference between the distance of the microsaccade end point from the memorized location and the distance of the microsaccade start point from that location. Negative values indicate a microsaccade toward the remembered location, positive values correspond to microsaccades away from the remembered location. During the fixation period, prior to the cue, microsaccade direction was not biased toward the future memory location (n=55 session-location combinations, microshift=0.025 ± 0.021, p=0.348; Supplementary Fig. 5a, gray histogram). Next we examined microsaccades during the memory period. The direction of microsaccades is biased toward the remembered location during the memory period (microshift=-0.056 ± 0.012, p=0.0004, n=55; Supplementary Fig. 5a, green histogram). To determine whether the occurrence of microsaccades caused any significant changes in the firing rate of MT neurons, and also to identify the specific time interval either before or after each microsaccade associated with these firing changes (if they occur), we measured a rate modulation index using sliding windows with different widths (ranging from 50 to 200 ms), starting at different times relative to the onset of microsaccades (ranging from 450 ms before each microsaccade onset to 400 ms after). The rate modulation was calculated as follows: rate modulation index=($FR_{sliding\ window}$ – $FR_{baseline}$)/ ($FR_{sliding\ window}$+ $FR_{baseline}$). The baseline firing rate is the firing rate during a time interval with the width of sliding window, starting 500 ms before each microsaccade. We found that during the memory period, the firing rate of neurons significantly increased after each microsaccade. The maximum rate modulation was observed for a window width of 150 ms starting from 90 ms after the microsaccade (n=425 neuron-location combinations, rate modulation=0.088 ± 0.019, p=0.00003, Supplementary Fig. 5b). Specifically, Supplementary Fig.5c shows the rate modulation of MT neurons measured with a sliding window with a width of 150 ms at different times relative to microsaccade onset. The bold portion shows the time period with significant rate modulation after microsaccade onset (p<0.05). Again, the maximum rate modulation is observed 90ms after the onset of microsaccades. This time interval is very similar to the time window reported for previous

microsaccade-associated firing rate modulations[13]. We then measured the rate modulation using this time interval (90-240 ms after each microsaccade, compared to a baseline 150-0 ms before each microsaccade in which we found no significant rate modulation (Supplementary Fig.5c, n=425 neuron-location combinations, rate modulation=0.025 ± 0.020, p=0.221)). During the memory period the overall rate modulation was significantly increased after microsaccades (n=425 neuron-location combinations, rate modulation=0.029 ± 0.010, p=0.037). We also found that microsaccades toward remembered locations are associated with greater rate modulation (Supplementary Fig. 5d, green plot; the negative values on x-axis indicates microsaccades toward a remembered location and the bold portion indicates significant rate modulation ($p<0.05$), Pearson correlation; r=-0.06, p=0.025). During fixation there was no overall rate modulation 90-240ms after microsaccades, and also no relationship between the direction of microsaccades and rate modulation (n=425 neuron-location combinations, rate modulation=0.006 ± 0.014, p=0.308, Supplementary Fig. 5d, gray plot, Pearson correlation; r=0.087, p=0.081).

Finally, we examined the relationship between microsaccades and noise correlations. During the fixation period, we see no change in noise correlation values before vs. after microsaccades (n=350 pairs, $\Delta \text{correlation}_{\text{after-before}}$=-0.014 ± 0.010, p=0.667; Supplementary Fig. 5e); nor was there any relationship between microshift direction and changes in noise correlation (Pearson correlation; r=-0.016, p=0.774). Then we asked whether the observed changes in noise correlation based on memory location were still significant when excluding the time period in which microsaccade-driven rate modulation occurred. When we exclude the period 90-240ms after any microsaccades from the noise correlation analysis, we still see an increase in noise correlations during memory at the Peak location for overlapping pairs (n=43 pairs; $\Delta \text{correlation}_{\text{Peak}}$=0.025 ± 0.010, p=0.016; Supplementary Fig. 5f blue), and a decrease in noise correlation during memory at the In-flank location for partially overlapping pairs (n=103 pairs, $\Delta \text{correlation}_{\text{In-flank}}$=-0.017 ± 0.005, p=0.001; Supplementary Fig. 5f red). Altogether, these results demonstrate that microsaccades are biased by WM, but do not drive the changes in noise correlations during WM.

## Supplementary Note 6

### The probe presentation paradigm did not alter saccade error in the MGS task

There was no effect of probe presence on memory accuracy. Performance was quantified using the average saccade error (difference between the saccade landing point and cue location), normalized based on target eccentricity (since larger errors are expected for more eccentric targets). There was no significant effect of the presence of visual probes on saccade error (Supplementary Fig. 6; saccade $\text{error}_{\text{with probes}}$=0.212 ± 0.012, n=54 session-memory condition combinations; saccade $\text{error}_{\text{without probes}}$=0.232 ± 0.009, n=65 session-memory condition combinations; with vs. without probes, p=0.106). There was no significant relationship between individual probe locations and saccade error (Pearson correlation; r=-0.012, p=0.568). There

was also no significant relationship between the mean probe location, in terms of distance from the remembered location, and saccade error (Pearson correlation; r=-0.044, p=0.114). There was a significant increase in reaction times when visual probes appeared during the delay (reaction $time_{with\ probes}$=209.131 ± 4.288 ms, n=54 session-memory conditions; reaction $time_{without\ probes}$=185.261 ± 2.463 ms, n=65 session-memory conditions, p=0.0001 Wilcoxon rank-sum test). There was no significant relationship between individual probe locations and reaction time (Pearson correlation; r=0.029, p=0.171). However, there was a significant relationship between the mean probe location, in terms of distance from the remembered location, and reaction time (Pearson correlation; r=0.113, $p<10^{-4}$). Overall, then, the presentation of probes slowed reaction times, and average probe locations farther from the memory location corresponded with larger reaction times, but there was no effect on memory accuracy.

## Supplementary Note 7

### Adaptation does not account for changes in noise correlations during WM

Repeated stimulus presentations can alter the firing rates of MT neurons17–19. We sought to determine whether recent stimulus history could likewise explain the observed changes in noise correlations in the visual responses of neuron pairs. First, we looked for evidence of an effect of the prior stimulus on probe-evoked responses in individual neurons. Since there were multiple probe locations inside vs. outside each neuron's RF, we used firing rate in response to the previous probe, rather than location, to divide trials. We divided trials for each visual probe into weak and strong groups based on the strength of the neuron's response to the previous visual probe (i.e. trials with a response higher or lower than the median of all responses were assigned to strong and weak trial groups, respectively). The response of each neuron was normalized to its maximum probe-evoked response. For preferred probes (probes evoking a greater than average response, i.e. closer to the RF center), stronger responses to the previous stimulus were associated with weaker responses to the same subsequent probe, consistent with an adaptation effect (Supplementary Fig. 10a). Probe-evoked firing rates were greater in weak trials compared to strong trials, and this effect was stronger for probes appearing closer to the peak of the RF (Pearson correlation; r=0.085, p<10-3; Supplementary Fig. 10b). Next, we looked for an effect of prior response history on noise correlations. Probe responses were once again divided into strong and weak groups based on the response to the previous probe; we measured the correlated variability of 121 overlapping pairs during the fixation period for weak vs. strong trials. First we confirmed the firing rate adaptation effect in individual neurons, as shown above, specifically for probes presented at the peak of the RF ($\Delta$normalized $FR_{weak-strong}$=0.051 ± 0.015, p=0.00039, n=85 neurons; Supplementary Fig. 10c). Next we compared the noise correlation values for neuron pairs in response to these peak-RF probes, and found that recent history has no effect on the noise correlation of overlapping pairs ($\Delta correlation_{weak-strong}$=0.006 ± 0.017, p=0.892, n=121 pairs; Supplementary Fig. 10d). We also confirmed that there is no significant difference between the noise correlations of pairs for either strong ($\Delta correlation_{strong-all}$=-0.004 ± 0.011, p=0.935, n=121 pairs; Supplementary Fig. 10e) or weak

($\Delta$correlation$_{weak\text{-}all}$=0.002 ± 0.008, p=0.753, n=121 pairs; Supplementary Fig. 10f) trials compared to all trials. We further looked at noise correlation values for weak vs. strong trials across probe locations. There is no difference between noise correlation of neurons during weak and strong trials ($\Delta$correlation$_{weak\text{-}strong}$ =0.004± 0.003, p=0.764, n=121 pairs), and, unlike the firing rate, no systematic effect of probe location on the difference in noise correlation between weak and strong trials (Pearson correlation; r=0.024, p=0.094). Finally, we examined whether partitioning the trials into weak and strong trials changes the main findings for the overlapping and partially overlapping pairs shown in figure 4d. We confirmed that there is no significant difference between the noise correlation changes for the Peak location of overlapping pairs if we consider either weak or strong trials ($\Delta$correlation$_{weak\text{-}strong}$=0.067 ± 0.148, p=0.840; $\Delta$correlation$_{weak\text{-}all}$=0.075 ± 0.077, p=0.193; $\Delta$correlation$_{strong\text{-}all}$=-0.051 ± 0.140, p=0.839; n=17 overlapping pairs). We also did not find a significant difference between the noise correlation changes for the In-flank location of partially overlapping pairs when we analyze either weak or strong trials ($\Delta$correlation$_{weak\text{-}strong}$=0.022 ± 0.134, p=0.961; $\Delta$correlation$_{weak\text{-}all}$=-0.006 ± 0.035, p=0.596; $\Delta$correlation$_{strong\text{-}all}$=-0.032 ± 0.133, p=0.731; n=56 partially overlapping pairs).This verifies that the adaptation and stimulus history are not contributing to the observed noise correlation changes.

## Supplementary Note 8

### A conceptual network model underlying the observed changes in noise correlations

Here we describe a set of plausible neural network mechanisms underlying the observed changes in correlated neural activity, schematically illustrated in Supplementary Fig. 12. We posit a spatially specific top-down excitatory input from FEF to MT[12,20,21]. In isolation, this common, top-down excitatory input will increase the correlation between visual neurons with overlapping RFs. The local inhibitory network illustrated in the figure is only active—or, alternatively, only active strongly enough to influence the excitatory network—in the presence of <u>both</u> bottom-up and top-down input; activity in this local inhibitory network produces a decorrelation among neurons with overlapping RFs. A combination of strong excitatory and inhibitory input has been reported to result in decorrelation among neurons despite their shared input[22,23]. To account for the changes in correlations between neurons with partially-overlapping RFs, a broader inhibitory network must also be present (not shown in figure). This model accounts for the changes in noise correlations we observed in the presence of top-down input with and without a visual stimulus, for pairs of neurons with overlapping or only partially-overlapping RFs. Top-down input only (memory IN vs. memory OUT, no visual stimulus): In the presence of top-down input only, this common excitatory input correlates the activity of neurons representing the same area of space. This top-down input decorrelates the activity of neurons with only partially-overlapping RFs via a broader lateral inhibitory network (not shown in the schematic because its location is uncertain); these longer-range inhibitory interactions could occur either within the target area (MT), or within the source of the top-down input (e.g. FEF). Top-down and bottom-up input (memory IN vs. memory OUT, with visual probe): In the presence

of both top-down and bottom-up visual input, the local inhibitory network is activated. This inhibitory signal causes decorrelation among neurons representing the same visual stimulus (overlapping pairs). We suggest that this local inhibitory network also inhibits the broader inhibitory network: for neurons with partially-overlapping RFs, the suppression of this broader inhibitory network removes its decorrelative effects, resulting in increased correlations between partially-overlapping neurons in the presence of both top-down and bottom-up input.

## Supplementary Discussion

### Relationship of these findings to attention literature

Many studies already link spatial attention and working memory. At a behavioral level, human psychophysical studies found visual perceptual benefits at locations held in WM, similar to the effect of covert visual attention[24]. Functional imaging studies have also reported increased visual cortical activity during WM tasks[25,26], again resembling the effects of covert spatial attention. Our own recent neurophysiological work demonstrated that extrastriate neurons representing a remembered location are more sensitive to incoming stimuli, and nearby neurons shift their RFs toward the remembered location[6]—confirming the resemblance between the effects of WM and attention at the level of individual neural responses in extrastriate cortex[27–30]. Since FEF neurons projecting to extrastriate cortex predominately contain WM-related delay activity[6], and this activity is related to attentional modulation within FEF[31], the neural mechanism of top-down modulation in the two cases may in fact be the same: top-down input from FEF neurons with delay activity projecting to visual areas. Moreover, we found that although the firing rate of neurons in visual areas does not change during the delay period of the MGS task, the content of WM is reflected in the LFP oscillations and spike timing. Interestingly, these changes were associated with an enhanced representation of sensory information presented at the locus of WM[7]. These spatially-specific changes in LFP oscillations and spike timing could be a basis for the noise correlation changes reported in this paper. LFP oscillations might drive both changes in spike synchrony which affect downstream signal transmission and changes in local correlations which impact the quality of the population representation; testing this theory may require optogenetics or other causal methods for manipulating synchronous activity.

It should be noted that our results in the presence of a visual stimulus are consistent with the findings of Ruff and Cohen in a covert attention task[10]. They report that attention can either increase or decrease noise correlations depending on a tuning similarity measure which reflects the extent of RF overlap between units. They see an increase in correlation for neurons with low tuning similarity (partially-overlapping RFs), and a decrease in correlations between neurons with high tuning similarity (overlapping RFs). A critical difference between their task and ours is the behavioral relevance of the visual stimulus—in their task, an explicit comparison of the contrast values of visual stimuli at different locations is the basis for task performance, and the observed increases and decreases in noise correlations are consistent with improving the neural basis for that comparison. This leaves open the possibility that attention-induced changes in

noise correlations arise from training and behavioral demands. Our finding that similar increases or decreases in noise correlations occur based on RF similarity even when the visual stimulus has no task or behavioral relevance suggests that such changes are instead innate, or at least the default effect of the top-down signal.

## Supplementary References


1. Squire, R. F., Noudoost, B., Schafer, R. J. & Moore, T. Prefrontal Contributions to Visual Selective Attention. *Annu. Rev. Neurosci.* **36,** 451–466 (2013).

2. Paneri, S. & Gregoriou, G. G. Top-Down Control of Visual Attention by the Prefrontal Cortex. Functional Specialization and Long-Range Interactions. *Front. Neurosci.* **11,** 545 (2017).

3. Clark, K. L., Noudoost, B., Schafer, R. J. & Moore, T. in *Handbook of Attention* (eds. Kastner, S. & Nobre, A. C.) (Oxford, 2014).

4. Clark, K., Squire, R. F., Merrikhi, Y. & Noudoost, B. Visual attention: Linking prefrontal sources to neuronal and behavioral correlates. *Prog. Neurobiol.* (2015). doi:10.1016/j.pneurobio.2015.06.006

5. Moore, T., Armstrong, K. M. & Fallah, M. Visuomotor origins of covert spatial attention. *Neuron* **40,** 671–83 (2003).

6. Merrikhi, Y. *et al.* Spatial working memory alters the efficacy of input to visual cortex. *Nat. Commun.* **8,** 15041 (2017).

7. Bahmani, Z., Daliri, M. R., Merrikhi, Y., Clark, K. & Noudoost, B. Working Memory Enhances Cortical Representations via Spatially Specific Coordination of Spike Times. *Neuron* **97,** 967–979.e6 (2018).

8. Postle, B. R. Working memory as an emergent property of the mind and brain. *Neuroscience* **139,** 23–38 (2006).

9. Mitchell, J. F., Sundberg, K. A. & Reynolds, J. H. Spatial attention decorrelates intrinsic activity fluctuations in macaque area V4. *Neuron* **63,** 879–88 (2009).

10. Ruff, D. A. & Cohen, M. R. Attention can either increase or decrease spike count correlations in visual cortex. *Nat. Neurosci.* **17,** 1591–7 (2014).

11. Herrero, J. L., Gieselmann, M. A., Sanayei, M. & Thiele, A. Attention-induced variance and noise correlation reduction in macaque V1 is mediated by NMDA receptors. *Neuron* **78,** 729–39 (2013).

12. Anderson, J. C., Kennedy, H. & Martin, K. A. C. Pathways of attention: synaptic relationships of frontal eye field to V4, lateral intraparietal cortex, and area 46 in macaque monkey. *J. Neurosci.* **31,** 10872–81 (2011).

13. Lowet, E. *et al.* Enhanced Neural Processing by Covert Attention only during Microsaccades Directed toward the Attended Stimulus. *Neuron* **99,** 207–214.e3 (2018).

14. Hafed, Z. M. Alteration of Visual Perception prior to Microsaccades. *Neuron* **77,** 775–786 (2013).

15. Chen, C.-Y., Ignashchenkova, A., Thier, P. & Hafed, Z. M. Neuronal Response Gain Enhancement prior to Microsaccades. *Curr. Biol.* **25,** 2065–74 (2015).

16. Hafed, Z. M. & Clark, J. J. Microsaccades as an overt measure of covert attention shifts. *Vision Res.* **42,** 2533–45 (2002).

17. Kohn, A. & Movshon, J. A. Neuronal adaptation to visual motion in area MT of the macaque. *Neuron* **39,** 681–91 (2003).

18. Petersen, S. E., Baker, J. F. & Allman, J. M. Direction-specific adaptation in area MT of the owl monkey. *Brain Res.* **346,** 146–50 (1985).

19. Van Wezel, R. J. A. & Britten, K. H. Motion Adaptation in Area MT. *J. Neurophysiol.* **88,** 3469–3476 (2002).

20. Stanton, G. B., Bruce, C. J. & Goldberg, M. E. Topography of projections to posterior cortical areas from the macaque frontal eye fields. *J. Comp. Neurol.* **353,** 291–305 (1995).

21. Schall, J. D., Morel, A., King, D. J. & Bullier, J. Topography of visual cortex connections with frontal eye field in macaque: convergence and segregation of processing streams. *J. Neurosci.* **15,** 4464–87 (1995).

22. Graupner, M. & Reyes, A. D. Synaptic Input Correlations Leading to Membrane Potential Decorrelation of Spontaneous Activity in Cortex. *J. Neurosci.* **33,** 15075–15085 (2013).

23. Renart, A. *et al.* The Asynchronous State in Cortical Circuits. *Science (80-. ).* **327,** 587–590 (2010).

24. Awh, E., Jonides, J. & Reuter-Lorenz, P. A. Rehearsal in Spatial Working Memory. *J. Exp. Psychol. Hum. Percept. Perform.* **24,** 780–790 (1998).

25. Corbetta, M., Kincade, J. M. & Shulman, G. L. Neural systems for visual orienting and their relationships to spatial working memory. *J. Cogn. Neurosci.* **14,** 508–23 (2002).

26. Postle, B. R., Awh, E., Jonides, J., Smith, E. E. & D'Esposito, M. The where and how of attention-based rehearsal in spatial working memory. *Brain Res. Cogn. Brain Res.* **20,** 194–205 (2004).

27. Womelsdorf, T., Anton-Erxleben, K. & Treue, S. Receptive field shift and shrinkage in macaque middle temporal area through attentional gain modulation. *J. Neurosci.* **28,** 8934–44 (2008).

28. Womelsdorf, T., Anton-Erxleben, K., Pieper, F. & Treue, S. Dynamic shifts of visual receptive fields in cortical area MT by spatial attention. *Nat. Neurosci.* **9,** 1156–60 (2006).

29. Niebergall, R., Khayat, P. S., Treue, S. & Martínez Trujillo, J. C. Expansion of MT Neurons Excitatory Receptive Fields during Covert Attentive Tracking. *J. Neurosci.* **31,** 15499–15510 (2011).

30. Treue, S. & Maunsell, J. H. Attentional modulation of visual motion processing in cortical areas MT and MST. *Nature* **382,** 539–41 (1996).

31. Armstrong, K. M., Chang, M. H. & Moore, T. Selection and maintenance of spatial information by frontal eye field neurons. *J. Neurosci.* **29,** 15621–9 (2009).
