# Supplementary material for: Concurrent influence of top-down and bottom-up inputs on correlated activity of Macaque extrastriate neurons
Source: Nat Commun. 2018 Dec 19;9:5393. doi: 10.1038/s41467-018-07816-4 (PMC6300596; doi:10.1038/s41467-018-07816-4)
Supplement: Supplementary file 2 — Peer Review [file 41467_2018_7816_MOESM2_ESM.docx]

Reviewers' comments:

Reviewer #1 (Remarks to the Author):

This manuscript presents interesting results about the effects of receptive field location and working memory demands on noise correlations between pairs of neurons. The noise correlations differ (in fact, can reverse) depending on the degree of RF overlap, whether a stimulus is the RF peak, and by WM demands. The analyses are fairly straightforward and the conclusions seem well-supported. 

General comments:

1. I am wondering why the authors limited themselves to just spiking activity. Local field potentials, especially as they relate to spiking, have proven to provide additional richness to investigations of network properties. This isn’t just a blanket statement. The authors raised the topic of top-down influences from the prefrontal cortex to dorsal streams areas and LFPs rhythms have been shown to correlate with such influences (e.g., see work by Fries and Womelsdorf, Buschman and Miller). The authors may say that this is beyond the scope of detailed RF properties and their relation to noise correlations but I think it is directly relevant. That work should be discussed in relation to this. If it were my dataset, I would have also studied the LFPs.

2. While the work relating noise correlation to RF overlap is detailed and well-done, I don’t see any reporting/discussion of the distance between neuron pairs. This will correlate with RF overlap, but not completely. It is a factor that directly affects their conclusions.

3-1. Many of the comparisons are between stimuli and WM locations at Peak or Flank locations relative to a location in the opposite hemifield. MT neuron RFs are heavily biased toward contralateral space. Thus, I think it relevant to see the same comparison between Peak/Flank etc locations relative to a location in the same hemifield but outside both neuron’s RF. A difference between this comparison and the comparison with the opposite hemifield would impact the authors’ conclusion and their model.

3-2. I may have missed it, but I saw discussion of overlapping RFs and partially overlapping RF, but nothing about non-overlapping RFs. Is that not relevant comparison? It would seem more complete to include such comparisons, even if only to confirm prior results.

4. RFs were mapped both during fixation and WM conditions. How did the RFs compare between these conditions? How did the WM requirement affect the visual responses?

5. The bump attractor model has been popular for explaining WM-related delay activity but there have been recent challenges to this model because of how easily memory maintenance in this model can be disrupted. It might be worth discussing alternative models and how the results fit with them.

Reviewer #2 (Remarks to the Author):

The manuscript titled “Concurrent influence of top-down and bottom-up inputs on correlated activity of Macaque extrastriate neurons” by Merrikhi and colleagues presents an interesting set of findings about the influence of spatial working memory on noise correlations in sensory cortex. Correlated fluctuations of activity across neural populations, noise correlations, have been of great interest in the study of neural coding and sensory processing recently because if they represent a source of internal noise they can limit the amount of information that can be read-out from a population. Thus modulation of noise correlations by cognitive factors, such as working memory or attention, may have important implications for the quality of sensory processing. Previous studies have shown that directing spatial attention to a location can cause a reduction in noise correlations among those neurons representing that location (Cohen and Maunsell, 2009; Mitchell et al, 2009). This study
finds that working memory directed to a spatial location in the absence of a stimulus causes an increase in noise correlations rather than a decrease, and that the increase is specific to pairs of neurons with overlapping receptive fields. Although this result may seem contradictory with previous results, it is consistent with some theoretical models that predict variability in top-down modulation underlie the fluctuations in noise correlations seen in sensory cortex (Ecker et al, 2016). The study finds that reduction of noise correlations is present when a sensory stimulus is present during the working memory task, which is broadly consistent with previous studies of attention. They expand on previous results to quantify how the effects vary for neuronal pairs with non-overlapping receptive fields and when the remembered locations fall to the side or outside receptive fields under study. The authors introduce a conceptual model that explains their findings through a non-linearity in inhibition, with inhibition having a minimal role when no stimulus is present but once elevated in baseline due to a stimulus driving a reduction in noise correlations under top-down feedback. 

Overall the results make a valuable contribution to the field and the manuscript is well written. However, it seems clear that the experimental design was not originally optimized to address the specific questions presented in the results. Instead, it appears that it would have been designed to examine how working memory influences receptive field structure and potential shifts in RF locations. These issues in design introduce complicating factors into the analysis of noise correlations that need to be addressed to insure the results are not confounded by other underlying causes. If those issues can be addressed without making the manuscript too complicated, it would be a valuable contribution.

I have two main concerns to address: 1) what is the potential role of modulation from small eye movements during the working memory task, and 2) what is the potential role of stimulus history in the flashing probe stimulus that could contribute some signal (adaptation history shared by neurons with overlapping RFs) into the firing rate of the currently flashed stimulus and thus contribute to noise correlations. I will briefly expand on these concerns.

First, recent studies have shown that small fixational eye movements can induce changes in neural activity of visual neurons in other brain areas (Chen et al, 2015; Hafed et al , 2015). The modulation from small eye movements also depends on their direction, and other studies have shown that during spatial attention fixational eye movements are directionally biased towards attended targets (Hafed and Clark, 2002). If such eye movements played a role in the current task during working memory for a spatial location, then they could cause a modulation that was specific towards the direction of the working memory target, which would coactivate neurons with overlapping receptive fields, increasing noise correlations. To address this concern, authors should flag those small eye movements that they are capable of measuring (probably those about 0.2 to 1 deg in amplitude that are within their fixation window) and time-locking on them analyze the firing rate modulation (if any) induced
by micro-saccades. They should also address if that modulation is direction specific (dependent on the direction of the micro-saccade) and to what extent such modulations might contribute to the noise correlations found, particularly for the case where no stimulus was present.

Second, the visual stimulus used in the second part of the study was a set of flashed probes randomly selected across locations on a 7x7 grid. The authors analyze noise correlations comparing epochs 30-230ms after a probe flashed at an identical location. Within that brief epoch the sensory stimulus is identical, but most likely the neuronal response will also depend on the neuron’s adaptation history and thus depend on the probe stimulus presented immediately before that one (even the stimulus presented two before that one), which would be at random locations. Further, neurons with overlapping receptive fields might share similar history dependence (as the random location stimulated for both cells before the current flash would likely either hit both or neither of them). Thus the authors need to quantify how much the neuronal response depends on the stimulus history before the current probe used for the noise correlations analysis, and try to address how much it contributes to the “noise” correlations in the analysis, or otherwise provide a convincing argument why that shared stimulus history could not contribute to the observed results.

Reviewer #3 (Remarks to the Author):

Merrikhi et al, Nat Comms.

The authors sought to evaluate how visual spatial WM maintenance altered the pairwise correlation between the spiking responses of simultaneously-recorded neurons in macaque area MT. Moreover, they were interested in whether this network activity structure was sculpted by bottom-up stimulation (when visual probes were presented during the memory delay period). They found that, depending on the spatial tuning similarity between two simultaneously-recorded neurons, spatial WM had opposing effects: when tuning similarity was high, WM increased spike count correlations; when cells shared partially-overlapping RFs, WM decreased spike count correlations. The opposite was observed when visual stimuli were presented during the delay period: similar-RF neuron pairs decreased their correlations, while partially-overlapping-RF pairs increased their correlations.  

I am very enthusiastic about this work, and find these data quite interesting. This report adds further evidence that even in the absence of changes in firing rates with WM, there are changes in activity patterns (noise correlations, spike timing, LFP) in sensory cortices. As such, I think this report would be of strong interest to the readership of Nature Communications. I have several comments the authors may wish to address:

Major:
1) Throughout the manuscript, the authors refer to their measures as 'noise correlations'. Because there are no changes in firing rate with WM maintenance in the absence of probe stimuli (Figs. 2&3, supported by Supp Fig. 3), this is the only type of correlation possible in this condition. However, when the probe stimuli are presented, the response of two simultaneously-measured neurons could be due to spontaneous activity fluctuations that are unrelated to the stimulus (noise correlations), or they could be due to shared activity related to the stimulus (signal correlations; discussed in Averbeck et al 2006, NRN). Perhaps this is accounted for in the rate matching procedure, or by breaking down responses as a function of tuning similarity, but I'm having trouble intuiting how. I don't necessarily believe this is a problem with the report, but I do think the exposition of the results could be clarified. If only spike count correlations are examined with no effort to address signal vs noise correlations, perhaps "spike count correlation" could replace "noise correlation" throughout most of the manuscript? Alternatively, if I'm misunderstanding part of the analysis which enables the authors to confidently attribute changes in variability to changes in noise correlations, additional discussion within the manuscript of this feature of the experiments/analyses would help avoid reader confusion.

2) Could the authors' previous excellent contribution to Nature Communications (Merrikhi et al, 2017, Nat Comms) provide a hint as to what is happening in the partial-overlap condition during visual stimulation? If I'm understanding this and the previous report correctly, neurons with partially-overlapping RFs will each extend their RFs to overlap a bit more if the WM position is placed within the 'flank' region of each neuron's RF. So, in Fig. 4c, we might imagine that the blue and green RFs (which I believe were measured during the fixation task, as described in Fig. 1a) will each reach down and to the right to be closer to location 1, larger, and their firing rate could increase (Merrikhi et al, 2017, Fig. 4). Wouldn't each of those RF changes contribute to an increase in spike count correlation? And would this be a result of an increase in signal correlation, or is there a further demonstrable role of changes in noise correlation? However, I'm not sure how such an explanation could account for the decrease in spike count correlation observed with overlapping RFs. In any case, I think this may be a point worthy of discussion - either addressing how the current results are supported by previous observations of RF profiles, or how the two results tell a different/complementary story.

3) To what extent is it fair to consider the RF mapping probes exogenous attention cues (e.g., Carrasco, 2011)? Do they have any impact on the animal's performance, either positive or negative? (for example, if the WM location itself is exactly 'probed', does that enhance the WM representation? Is there bias induced by the mean probe location in the memory guided saccade position?). It doesn't seem possible to measure visual responses during a WM task without some sort of induction of exogenous attention, but I do wonder what the authors think about the role of exogenous attention in their task. In general, further exposition of behavioral performance on the MGS task would be useful, as would exploration into whether the observed neural results are manifest in changes in behavioral performance.

4) Figure 5 is very hard to interpret. I can't parse what's meant to be shown in panel a (what are the 4 bars?), and panel b is similarly a bit confusing. The model in principle makes sense, but it's very challenging to intuit how the tuning profiles drawn in panel b support changes in correlations. Most of the confusion I think comes from the (I believe?) fact that the inhibitory input should only be activated in presence of visual input, but that's not easy to intuit from the figure. The authors may wish to try to clarify this illustration.

Minor:

1) Pg 21 - What color were the square targets for the MGS task? And why is the target in Fig. 1 a circle?

2) MGS task - a +/- 4 dva response window seems very large - how precise were the animals in their response? Were they closer to the actual target location, or to the other possible locations, which seem to be < 4 dva apart (Fig. 1c, at least for some sessions).

3) For the RF mapping task (pg 20), it seems as though the responses to all probes were used. However, for the memory guided saccade task with visual probes (pg 21-22), the first probe was dropped (lines 495-496) - why was this?

4) The authors should include a data/code availability statement (this is mentioned in the checklist, but I cannot find it in the manuscript). I encourage the authors to deposit data sufficient to reproduce the results reported in the manuscript and associated analysis code on a public repository like OSF

5) Figs 2 & 4 - for the single-neuron examples, the authors use a shuffling procedure to evaluate significance - could they elaborate on this procedure in the methods? Is this applied to all neurons and only significant neurons included in further analyses? This does not appear to be the case, but it could be useful to clarify. Additionally, the authors could include a measure of variability in the single-neuron bar graphs - perhaps a gray band or dashed line near each bar marking the confidence interval of the shuffled 'null' distribution?

6) The scatterplots in Figs 3 and 4 should be better labeled, and the remaining cases also shown (either in a separate panel or as unfilled circles). For example, at present, Fig. 3a shows, in blue, correlation coefficients for pairs of neurons with overlapping RFs and WM location in their shared peak, and in red partially-overlapping pairs of neurons with a WM location in their flanks. There are other cases, though, like overlapping pairs with WM location in flanks, partially-overlapping pairs with WM location in one or another peak, etc. I understand the two displayed conditions are the most salient of the report, but if there's a way to show the entire dataset more clearly, that would be ideal. I believe this information may be found in the heatmap in Fig 3b and the line graphs in Fig. 4d-e, but those do not show individual data points.

7) Fig. 4b-c - are the RF contours in these schematics computed from the RF mapping trials (no WM) or from the MGS task with visual probes (Fig. 4a) trials? I imagine both could be computed for these pairs to address the signal correlation point made above, and to allude to results reported previously in Merrikhi et al, 2017

8) Fig. 4d-e could be more clearly labeled - if I'm understanding correctly, the x axis is the mean response to the probe given a constant WM location? If so, perhaps "geometric mean of normalized probe responses" would be an appropriate x axis label? And I'm also having trouble understanding the relationship between WM location and probe location - where is the WM location relative to the probe? Is it any of the 4 "in" locations?

9) Supplementary Figs. 4 & 5 are informative, but very challenging to parse - I'd recommend adding labels to the figures above panels a, c, e indicating that these correspond to trials in which the animal is remembering in the peak, flanks, or tails of the pair, and a super-title over each figure labeling Supp Fig. 4 as showing results from overlapping and Supp Fig. 5 as showing results from partially-overlapping pairs.

Reviewers' comments:

Reviewer #1 (Remarks to the Author):

This manuscript presents interesting results about the effects of receptive field location and working memory demands on noise correlations between pairs of neurons. The noise correlations differ (in fact, can reverse) depending on the degree of RF overlap, whether a stimulus is the RF peak, and by WM demands. The analyses are fairly straightforward and the conclusions seem well-supported. 

General comments:
1. I am wondering why the authors limited themselves to just spiking activity. Local field potentials, especially as they relate to spiking, have proven to provide additional richness to investigations of network properties. This isn’t just a blanket statement. The authors raised the topic of top-down influences from the prefrontal cortex to dorsal streams areas and LFPs rhythms have been shown to correlate with such influences (e.g., see work by Fries and Womelsdorf, Buschman and Miller). The authors may say that this is beyond the scope of detailed RF properties and their relation to noise correlations but I think it is directly relevant. That work should be discussed in relation to this. If it were my dataset, I would have also studied the LFPs.

We agree with the reviewer that understanding how LFPs change during WM is extremely important. Our analysis of LFP changes during WM were submitted as a separate manuscript prior to this paper, and during the review process of the current paper they were published (Bahmani et al. Neuron 2018). As reported there, remembering a location in the RF of the recording site altered LFP power in the alpha-beta range, and increased spike-phase locking in the same frequency band. Now we have referred to these results in the introduction (page 2), Supplementary Information (page 6), and discussion section (page 17):

*“Recent findings from our lab suggest a mechanism within visual cortex that can serve as a common source of modulation. WM causes a spatially specific increase in the power of αβ oscillations in visual areas^15^. The degree to which neurons’ spike times lock to these oscillations also depends on the overlap between the neurons’ RF and the WM location^15^. Such changes in spike timing relative to a common oscillatory signal, when measured at an appropriate time scale across trials, affects correlated activity. Interestingly, in the presence of visual information, the spike-phase locking was altered based on the properties of the visual stimulus, potentially reducing the correlated activity. Changes in oscillatory properties within the visual area or in the top-down signal could both contribute to the observed changes in correlated activity.”* (page 17)

*“Moreover, we found that although the firing rate of neurons in visual areas does not change during the delay period of the MGS task, the content of WM is reflected in the LFP oscillations and spike timing. Interestingly, these changes were associated with an enhanced representation of sensory information presented at the locus of WM7. These spatially-specific changes in LFP oscillations and spike timing could be a basis for the noise correlation changes reported in this paper. LFP oscillations might drive both changes in spike synchrony which affect downstream signal transmission and changes in local correlations which impact the quality of the population representation; testing this theory may require optogenetics or other causal methods for manipulating synchronous activity.”* (Supplementary page 6)

2. While the work relating noise correlation to RF overlap is detailed and well-done, I don’t see any reporting/discussion of the distance between neuron pairs. This will correlate with RF overlap, but not completely. It is a factor that directly affects their conclusions.

We thank the reviewer for suggesting this control, as it is important to determine whether RF overlap or physical proximity between neurons is what drives their correlated variability. We now include an evaluation of the effect of cortical distance on page 4 of the text and first page of the Supplementary information.

Page 4 of the text:

*“RF overlap was a better predictor of noise correlations than physical distance between neurons (Supplementary Fig. 2).”*

First page of the Supplementary information:

*“It is important to establish whether the relationship between noise correlation and RF overlap is truly dependent on the RF similarity, or instead a secondary consequence of the relationship between RF overlap and cortical distance. As expected given the retinotopic organization of MT cortex, there is a correlation between cortical distance (measured as the distance between electrode contacts on which neurons were recorded) and RF overlap values for neuron pairs (r=-0.378, p<10^-12^). Accordingly, there is an inverse correlation between the noise correlation and the physical distance between the contacts where neurons were recorded: as the distance between electrodes increases the correlated variability of neurons decreases (Pearson correlation, r=-0.15 p=0.005; Supplementary Fig. 2). Importantly, however, a multi-variable linear regression factoring in both physical distance and RF overlap indicates that RF overlap is the crucial factor in predicting noise correlations: the noise correlation between neurons is dependent on the degree of overlap between their spatial RFs (r=0.140, p<10^-3^, linear regression), but not the distance between electrodes (r=4×10^-6^, p=0.751, linear regression; no significant interaction between spatial tuning and distance: r=-2×10^-5^, p=0.441, linear regression). Thus RF overlap, rather than feature tuning or physical distance, is the best predictor of noise correlations during fixation.”*

3-1. Many of the comparisons are between stimuli and WM locations at Peak or Flank locations relative to a location in the opposite hemifield. MT neuron RFs are heavily biased toward contralateral space. Thus, I think it relevant to see the same comparison between Peak/Flank etc locations relative to a location in the same hemifield but outside both neuron’s RF. A difference between this comparison and the comparison with the opposite hemifield would impact the authors’ conclusion and their model.

We have performed this control as suggested, and find that the main results (an increase in noise correlation for overlapping pairs, and a reduction in noise correlation for partially overlapping pairs) are also true for a comparison to a location in the same hemifield but outside both neuron’s RFs. We have added these results to Figure 3C, and the results section (page 9):

*“The same main effects (an increase in the correlation of overlapping pairs when remembering a Peak location, a decrease in the correlation of partially overlapping pairs when remembering an In-flank location) were seen when comparing Peak or In-flank memory conditions to a memory cue outside both neurons’ RFs, but within the same visual hemifield. For overlapping RF pairs, remembering a Peak location increases the correlated variability of neurons compared to a location in same hemifield but outside of both RFs (n=43 pairs, ∆correlationPeak=0.019 ± 0.009 , p=0.039; Fig. 3c, blue). For partially overlapping RF pairs, remembering an In-flank location decreases the correlated variability of neurons compared to a location in same hemifield but outside of both RFs (n=103 pairs, ∆correlationIn-flank=-0.012 ± 0.005 , p=0.017; Fig. 3c, red). Moreover, the effects for same-hemifield comparisons are not significantly different in magnitude than those observed for the opposite hemifield comparisons (∆correlationPeak=0.000 ± 0.008, p=0.771, n=43 pairs; ∆correlationIn-flank=0.006 ± 0.006, p=0.128, n=103 pairs). This control analysis reemphasizes the spatial dependence of changes in correlations, and suggests that the spatial extent of changes in correlated activity is probably on the order of RF size.”*

3-2. I may have missed it, but I saw discussion of overlapping RFs and partially overlapping RF, but nothing about non-overlapping RFs. Is that not relevant comparison? It would seem more complete to include such comparisons, even if only to confirm prior results.

There were no significant changes; we added this information to the Supplementary Information, page 3:

*“Non-overlapping pairs (RF ratio <0.001) showed no significant changes in noise correlation in the absence of visual probes, for any memory location (∆correlationPeaks=0.016 ± 0.014, p=0.5, n=2 pairs; ∆correlationIn-flank=-0.018 ± 0.015, p=0.301, n=9 pairs; ∆correlationOut-flanks=-0.001 ± 0.009, p=0.952, n=14 pairs). In the presence of visual probes, non-overlapping pairs also showed no significant changes in noise correlation for memory in the Peaks location, in the presence of visual probes at any location (∆correlationPeaks=0.118 ± 0.061, p=0.151, n=32 pair-probe; ∆correlationIn-flank=0.095 ± 0.099, p=0.391, n=20 pair-probe; ∆correlationOut-flanks=0.027 ± 0.047, p=0.757, n=37 pair-probe), nor for memory in the Out-flank location, in the presence of visual probes at any location (∆correlationPeaks=0.016 ± 0.039, p=0.280, n=70 pair-probe; correlationIn-flank=-0.089 ± 0.052, p=0.103, n=41 pair-probe; ∆correlationOut-flanks=0.001 ± 0.022, p=0.901, n=235 pair-probe). (There were not any non-overlapping pairs with the memory location at the In-flank location in the visual probe dataset.)“*

4. RFs were mapped both during fixation and WM conditions. How did the RFs compare between these conditions? How did the WM requirement affect the visual responses?

RFs expanded and shifted toward the remembered location, as reported in our previous work (Merrikhi et al. 2017). We present these results in Supplementary Fig. 11, and present the associated statistics on page 13 of the text:

*“In this dataset, there are 59 MT neurons with a well-shaped RF contour during the three memory IN conditions. For this set of neurons we found that during memory maintenance, the RF of neurons shifted (n=59, ∆distance_memory-baseline_=-0.230 ± 0.067, p=0.002) and expanded (∆RF diameter_memory-baseline_=0.288 ± 0.131, p=0.005) toward the remembered location (Supplementary Fig. 8). The gain of visual responses also increased (∆max response_memory-baseline_=3.239 ± 0.450 Hz, p<10^-6^).”*

We also examine the relationship of these RF changes to the changes in noise correlations. See Fig. 5 and response to reviewer 3 point 2.

5. The bump attractor model has been popular for explaining WM-related delay activity but there have been recent challenges to this model because of how easily memory maintenance in this model can be disrupted. It might be worth discussing alternative models and how the results fit with them.

That is correct. In general, any model of working memory which involves spatially specific persistent activity (as has been shown to exist within the FEF for the type of memory guided saccade task used in these experiments) would be consistent with our data; we have revised the wording of our discussion to reflect this (page 17):

“*The changes in noise correlations observed based on WM location and RF overlap in the absence of visual input (Figs 2 & 3) are consistent with an excitatory, top-down input from PFC reflecting spatially-specific WM activity of the sort which is known to exist in FEF^16,33–35^ and dlPFC^36–39^ during this task.”*

Models of working memory which do NOT involve persistent activity (e.g., activity-silent or synaptic theories of WM) do not obviously predict the results we report here—although some ‘activity-silent’ models involve periodic activity to refresh the synaptic mechanisms, which if averaged over longer timescales could produce a similar effect on measured noise correlations. Since we know that persistent activity does exist in the task used in our dataset, we don’t think our results make any clear claims or predictions regarding activity-silent models of WM.

Reviewer #2 (Remarks to the Author):

The manuscript titled “Concurrent influence of top-down and bottom-up inputs on correlated activity of Macaque extrastriate neurons” by Merrikhi and colleagues presents an interesting set of findings about the influence of spatial working memory on noise correlations in sensory cortex. Correlated fluctuations of activity across neural populations, noise correlations, have been of great interest in the study of neural coding and sensory processing recently because if they represent a source of internal noise they can limit the amount of information that can be read-out from a population. Thus modulation of noise correlations by cognitive factors, such as working memory or attention, may have important implications for the quality of sensory processing. Previous studies have shown that directing spatial attention to a location can cause a reduction in noise correlations among those neurons representing that location (Cohen and Maunsell, 2009; Mitchell et al, 2009). This study
finds that working memory directed to a spatial location in the absence of a stimulus causes an increase in noise correlations rather than a decrease, and that the increase is specific to pairs of neurons with overlapping receptive fields. Although this result may seem contradictory with previous results, it is consistent with some theoretical models that predict variability in top-down modulation underlie the fluctuations in noise correlations seen in sensory cortex (Ecker et al, 2016). The study finds that reduction of noise correlations is present when a sensory stimulus is present during the working memory task, which is broadly consistent with previous studies of attention. They expand on previous results to quantify how the effects vary for neuronal pairs with non-overlapping receptive fields and when the remembered locations fall to the side or outside receptive fields under study. The authors introduce a conceptual model that explains their findings through a non-linearity in inhibition, with inhibition having a minimal role when no stimulus is present but once elevated in baseline due to a stimulus driving a reduction in noise correlations under top-down feedback. 

Overall the results make a valuable contribution to the field and the manuscript is well written. However, it seems clear that the experimental design was not originally optimized to address the specific questions presented in the results. Instead, it appears that it would have been designed to examine how working memory influences receptive field structure and potential shifts in RF locations. These issues in design introduce complicating factors into the analysis of noise correlations that need to be addressed to insure the results are not confounded by other underlying causes. If those issues can be addressed without making the manuscript too complicated, it would be a valuable contribution.

I have two main concerns to address: 1) what is the potential role of modulation from small eye movements during the working memory task, and 2) what is the potential role of stimulus history in the flashing probe stimulus that could contribute some signal (adaptation history shared by neurons with overlapping RFs) into the firing rate of the currently flashed stimulus and thus contribute to noise correlations. I will briefly expand on these concerns.

First, recent studies have shown that small fixational eye movements can induce changes in neural activity of visual neurons in other brain areas (Chen et al, 2015; Hafed et al , 2015). The modulation from small eye movements also depends on their direction, and other studies have shown that during spatial attention fixational eye movements are directionally biased towards attended targets (Hafed and Clark, 2002). If such eye movements played a role in the current task during working memory for a spatial location, then they could cause a modulation that was specific towards the direction of the working memory target, which would coactivate neurons with overlapping receptive fields, increasing noise correlations. To address this concern, authors should flag those small eye movements that they are capable of measuring (probably those about 0.2 to 1 deg in amplitude that are within their fixation window) and time-locking on them analyze the firing rate modulation (if any) induced
by micro-saccades. They should also address if that modulation is direction specific (dependent on the direction of the micro-saccade) and to what extent such modulations might contribute to the noise correlations found, particularly for the case where no stimulus was present.

The reviewer is correct to say that this is an important control, especially in light of recent results (Lowet et al. 2018) showing a critical role for microsaccades in attentional modulation. We would have liked to include this control in the main text but were precluded by space limitations, and so it is presented in the Supplementary Information. Our analysis indicates that in our dataset microsaccades are biased by WM location and associated with rate modulation during the delay period, but not associated with changes in the noise correlations. We include the details of these analyses on page 2-3 of the Supplementary Information and Supplementary Figure 5:

*“In light of recent results emphasizing the importance of microsaccades during covert attentional modulations of extrastriate visual cortex^13–16^, we investigated what role, if any, microsaccades played in driving the changes in noise correlations during WM. For each microsaccade we calculated the microshift, defined as the difference between the distance of the microsaccade end point from the memorized location and the distance of the microsaccade start point from that location. Negative values indicate a microsaccade toward the remembered location, positive values correspond to microsaccades away from the remembered location. We also measured the rate modulation during the 100-200 ms after each microsaccade, compared to the 100ms before each saccade (rate modulation index=(FR_after_ - FR_before_)/(FR_after_ + FR_before_)). During the fixation period, prior to the cue, microsaccade direction was not biased toward the future memory location (n=55 session-location combinations, microshift=0.025 ± 0.021, p=0.348; Supplementary Fig. 5a, gray histogram). Also, there was no rate modulation after microsaccades during fixation (n=425 neuron-location combinations, rate modulation=-0.001 ± 0.015, p=0.762), and no relationship between the direction of the microsaccade and the rate modulation (Pearson correlation; r=-0.012, p=0.802; Supplementary Fig. 5b, gray plot). Next we examined microsaccades during the memory period. The direction of microsaccades is biased toward the remembered location during the memory period (microshift=-0.056 ± 0.012, p<0.001, n=55; Supplementary Fig. 5a, green histogram). Although there was no overall increase in firing rate after microsaccades (rate modulation=0.032 ± 0.011, p=0.080), there was a relationship between the direction of microsaccades and the rate modulation: microsaccades toward the remembered location were associated with an increase in firing rate (Pearson correlation; r=-0.098, p=0.046, Supplementary Fig. 5b, green plot).*

*Finally, we examine the relationship between microsaccades and noise correlations. During the fixation period, we see no change in noise correlation values before vs. after microsaccades (n=350 pairs, ∆correlation_after-before_=-0.013 ± 0.006, p=0.388; Supplementary Fig. 5c); nor was there any relationship between microshift direction and changes in noise correlation (Pearson correlation; r=-0.001, p=0.962). When we exclude the period 100-200ms after any microsaccades from the noise correlation analysis, we still see an increase in noise correlations during memory at the Peak location for overlapping pairs (n=43 pairs; ∆correlation_Peak_=0.021 ± 0.009, p=0.025; Supplementary Fig. 5d blue), and a decrease in noise correlation during memory at the In-flank location for partially overlapping pairs (n=103 pairs, ∆correlation_In-flank_=-0.017 ± 0.005, p=0.001; Supplementary Fig. 5d red). The 100-200 ms time window was chosen based on the time window for previous microsaccade-associated firing rate modulations^13^. For further analysis we also selected the 0-100ms time window after each microsaccade and repeated all the analyses. There was no significant relationship between the direction of microsaccades and the rate modulation during delay period (Pearson correlation; r=0.022, p=0.649). After excluding the period 0-100 ms after any microsaccades from the noise correlation analysis, we still found an increase in noise correlation for the Peak location of overlapping pairs (n=43 pairs, ∆correlation_Peak_=0.020 ± 0.009, p=0.034), and a decrease in noise correlation for the In-flank location of partially overlapping pairs (n=103 pairs; ∆correlation_In-flank_=-0.018 ± 0.005, p=0.001). Altogether, these results demonstrate that microsaccades are biased by WM, but do not drive the changes in noise correlations during WM.”*

Second, the visual stimulus used in the second part of the study was a set of flashed probes randomly selected across locations on a 7x7 grid. The authors analyze noise correlations comparing epochs 30-230ms after a probe flashed at an identical location. Within that brief epoch the sensory stimulus is identical, but most likely the neuronal response will also depend on the neuron’s adaptation history and thus depend on the probe stimulus presented immediately before that one (even the stimulus presented two before that one), which would be at random locations. Further, neurons with overlapping receptive fields might share similar history dependence (as the random location stimulated for both cells before the current flash would likely either hit both or neither of them). Thus the authors need to quantify how much the neuronal response depends on the stimulus history before the current probe used for the noise correlations analysis, and try to address how much it contributes to the “noise” correlations in the analysis, or otherwise provide a convincing argument why that shared stimulus history could not contribute to the observed results.

We thank the reviewer for another good suggestion. We find adaptation effects of firing rate based on recent history (firing rates in response to the previous stimulus impact responses to the subsequent probe); however, we do not find any evidence that the response to previous probes influences noise correlations. Details of the analysis are provided on page 4-5 of the Supplementary Information and in Supplementary Figure 10:

*“Repeated stimulus presentations can alter the firing rates of MT neurons^17–19^. We sought to determine whether recent stimulus history could likewise explain the observed changes in noise correlations in the visual responses of neuron pairs. First, we looked for evidence of an effect of the prior stimulus on probe-evoked responses in individual neurons. Since there were multiple probe locations inside vs. outside each neuron’s RF, we used firing rate in response to the previous probe, rather than location, to divide trials. We divided trials for each visual probe into ‘weak’ and ‘strong’ groups based on the strength of the neuron’s response to the previous visual probe (i.e. trials with a response higher or lower than the median of all responses were assigned to strong and weak trial groups, respectively). The response of each neuron was normalized to its maximum probe-evoked response. For preferred probes (probes evoking a greater than average response, i.e. closer to the RF center), stronger responses to the previous stimulus were associated with weaker responses to the same subsequent probe, consistent with an adaptation effect (Supplementary Fig. 10a). Probe-evoked firing rates were greater in weak trials compared to strong trials, and this effect was stronger for probes appearing closer to the peak of the RF (Pearson correlation; r=0.085, p<10^-3^; Supplementary Fig. 10b). Next, we looked for an effect of prior response history on noise correlations. Probe responses were once again divided into strong and weak groups based on the response to the previous probe; we measured the correlated variability of 121 overlapping pairs during the fixation period for weak vs. strong trials. First we confirmed the firing rate adaptation effect in individual neurons, as shown above, specifically for probes presented at the peak of the RF (∆normalized FR_weak-strong_=0.051 ± 0.015, p=0.00039, n=85 neurons; Supplementary Fig. 10c). Next we compared the noise correlation values for neuron pairs in response to these peak-RF probes, and found that recent history has no effect on the noise correlation of overlapping pairs (∆correlation_weak-strong_=0.006 ± 0.017, p=0.892, n=121 pairs; Supplementary Fig. 10d). We further looked at noise correlation values for weak vs. strong trials across probe locations. There is no difference between noise correlation of neurons during weak and strong trials (∆correlation _weak-strong_ =0.004± 0.003, p=0.764, n=121 pairs), and, unlike the firing rate, no systematic effect of probe location on the difference in noise correlation between weak and strong trials (Pearson correlation; r=0.024, p=0.094). In summary, our results show that previous stimulus history impacts probe-evoked firing rates but not pairwise noise correlations during WM.”*

Reviewer #3 (Remarks to the Author):

Merrikhi et al, Nat Comms.

The authors sought to evaluate how visual spatial WM maintenance altered the pairwise correlation between the spiking responses of simultaneously-recorded neurons in macaque area MT. Moreover, they were interested in whether this network activity structure was sculpted by bottom-up stimulation (when visual probes were presented during the memory delay period). They found that, depending on the spatial tuning similarity between two simultaneously-recorded neurons, spatial WM had opposing effects: when tuning similarity was high, WM increased spike count correlations; when cells shared partially-overlapping RFs, WM decreased spike count correlations. The opposite was observed when visual stimuli were presented during the delay period: similar-RF neuron pairs decreased their correlations, while partially-overlapping-RF pairs increased their correlations.  

I am very enthusiastic about this work, and find these data quite interesting. This report adds further evidence that even in the absence of changes in firing rates with WM, there are changes in activity patterns (noise correlations, spike timing, LFP) in sensory cortices. As such, I think this report would be of strong interest to the readership of Nature Communications. I have several comments the authors may wish to address:

Major:
1) Throughout the manuscript, the authors refer to their measures as 'noise correlations'. Because there are no changes in firing rate with WM maintenance in the absence of probe stimuli (Figs. 2&3, supported by Supp Fig. 3), this is the only type of correlation possible in this condition. However, when the probe stimuli are presented, the response of two simultaneously-measured neurons could be due to spontaneous activity fluctuations that are unrelated to the stimulus (noise correlations), or they could be due to shared activity related to the stimulus (signal correlations; discussed in Averbeck et al 2006, NRN). Perhaps this is accounted for in the rate matching procedure, or by breaking down responses as a function of tuning similarity, but I'm having trouble intuiting how. I don't necessarily believe this is a problem with the report, but I do think the exposition of the results could be clarified. If only spike count correlations are examined with no effort to address signal vs noise correlations, perhaps "spike count correlation" could replace "noise correlation" throughout most of the manuscript? Alternatively, if I'm misunderstanding part of the analysis which enables the authors to confidently attribute changes in variability to changes in noise correlations, additional discussion within the manuscript of this feature of the experiments/analyses would help avoid reader confusion.

All correlations with a visual stimulus are calculated across responses to the same visual stimulus (i.e., same probe location); thus these correlations are still noise correlations, looking at shared cross-trial variability in the response to an identical visual probe. We have added the following to the Methods section (page 25) to clarify this point:

*“In the case of visual probes, correlation coefficients were calculated based on the cross-trial variability in the visual response to a single probe presentation (30-230 ms following onset of a probe at the same location for all trials).”*

If the reviewer is concerned about the effects of stimulus history, which could be considered a type of signal correlation, see the analysis of history effects presented in the Supplementary Fig. 10 in response to reviewer #2, which indicates recent history does not impact the correlations.

2) Could the authors' previous excellent contribution to Nature Communications (Merrikhi et al, 2017, Nat Comms) provide a hint as to what is happening in the partial-overlap condition during visual stimulation? If I'm understanding this and the previous report correctly, neurons with partially-overlapping RFs will each extend their RFs to overlap a bit more if the WM position is placed within the 'flank' region of each neuron's RF. So, in Fig. 4c, we might imagine that the blue and green RFs (which I believe were measured during the fixation task, as described in Fig. 1a) will each reach down and to the right to be closer to location 1, larger, and their firing rate could increase (Merrikhi et al, 2017, Fig. 4). Wouldn't each of those RF changes contribute to an increase in spike count correlation? And would this be a result of an increase in signal correlation, or is there a further demonstrable role of changes in noise correlation? However, I'm not sure how such an explanation could account for the decrease in spike count correlation observed with overlapping RFs. In any case, I think this may be a point worthy of discussion - either addressing how the current results are supported by previous observations of RF profiles, or how the two results tell a different/complementary story.

This is an important factor to consider—given that in baseline conditions pairs with greater RF overlap show greater noise correlations, then changes in RF position or size during the memory period which result in greater overlap might be expected to increase the noise correlation between those neurons. (As the reviewer points out, this is a potential confound for the partially overlapping pairs, but wouldn’t seem to explain the results for overlapping pairs.) Here we show that 1) these RF shifts and expansions during the delay do not result in a greater RF overlap ratio for the partially overlapping pairs in our dataset, and 2) there is no correlation between change in RF overlap and change in noise correlation across pairs. Details of these analyses are presented on page 14-16 of the text and Figure 5:

*“In order to examine the relationship between changes in RF overlap and changes in correlated activity, we focused on pairs of neurons with partially overlapping RFs during the fixation period, since RF changes are more prominent when the WM locus is slightly off the center of the RF^14^. Figure 5a shows an example of how three MT neurons change their RF profile during the memory period. Consistent with our previous report^14^, the two neurons (red and blue) with RFs closer to the memorized location shift their RF toward that location during memory. The third neuron (green), a bit farther from that location, does not change its RF profile. Consequently, these changes can result in either an increase (between red and blue neurons) or decrease (between e.g. green and blue neurons) in the RF overlap between neurons, depending on how they change their RF profile during memory.*

*Out of 56 partially overlapping neuron pairs with the memory target presented at an In-flank location, we could obtain a well-shaped RF contour during the memory period for both neurons in 48 pairs (51 neurons). As expected, the RFs of these neurons shifted toward the remembered location (Fig. 5b bottom; n=51 neurons, ∆RF distance_memory-fixation_ =-0.414 ± 0.168, p=0.006) and expanded (Fig. 5b top; n=51 neurons, ∆RF size_memory-fixation_ =0.459 ± 0.229, p=0.014). Figure 5c shows the shared RF ratio of 48 pairs of neurons during fixation and memory, where partially overlapping pairs are defined as those with shared RF ratios between 0.1 and 0.4. The 31 pairs of neurons for which the partially overlapping definition is met during fixation and memory are shown in black in Fig. 5c&e. Overall, these shifts and expansions resulted in some changes in RF overlap, although without any specific direction over the population (n=48 pairs, ∆shared RF ratio_Inflank-fixation_=-0.003 ± 0.021, p=0.766; Figure 5d, bottom histogram). In spite of the lack of a systematic change in RF overlap, we still looked for a relationship between the change in shared RF ratio and the effect of memory on the noise correlation. The top panel in figure 5d shows the noise correlation for memory IN (red) and OUT (blue), as a function of the change in shared RF ratio. During the memory IN condition, noise correlations are actually higher for pairs which have decreased their RF overlap—the opposite of what one would expect if changes in RF overlap were driving the noise correlations (Pearson correlation; r=-0.49, p=0.0003). The increase in noise correlation between the memory IN and OUT conditions is independent of the change in RF overlap (Pearson correlation; r=-0.23, p=0.116). These results indicate that the changes in RF profiles do not induce a systematic change in the RF overlap in the current dataset; furthermore, in general an increase in RF overlap during memory corresponded with a decrease in the correlated activity, similar to effects of sharing a top-down signal (Fig. 4) and opposite to the effects of sharing a bottom signal (Supplementary figure 1).*

*We also performed a simpler control by using an even stricter criteria for identifying partially overlapping pairs and In-flank locations, limiting our analysis to pairs and locations which fit the definitions during both fixation and memory. For all 48 partially overlapping pairs, the noise correlation during In-flank memory is significantly higher than when remembering a location in opposite hemifield (Fig. 5e, all points; n=48 pairs, ∆correlation_In-flank_=0.068 ± 0.019, p=0.012). For the 31 pairs of neurons meeting the partially overlapping definition during both fixation and memory, there was still an increase in noise correlations when remembering the In-flank location compared to the location in opposite hemifield (Fig. 5e, black; n=31 pairs; ∆Correlation _In-flank_=0.08 ± 0.029, p=0.041). Thus although RF changes do occur during the memory period, the increase in noise correlation for partially overlapping pairs remains significant even when excluding pairs whose overlap category or sensitivity to the memory location has changed.”*

3) To what extent is it fair to consider the RF mapping probes exogenous attention cues (e.g., Carrasco, 2011)? Do they have any impact on the animal's performance, either positive or negative? (for example, if the WM location itself is exactly 'probed', does that enhance the WM representation? Is there bias induced by the mean probe location in the memory guided saccade position?). It doesn't seem possible to measure visual responses during a WM task without some sort of induction of exogenous attention, but I do wonder what the authors think about the role of exogenous attention in their task. In general, further exposition of behavioral performance on the MGS task would be useful, as would exploration into whether the observed neural results are manifest in changes in behavioral performance.

This information is now included on page 4 of the Supplementary Information and the Supplementary Fig. 6:

*“There was no effect of probe presence on memory accuracy. Performance was quantified using the average saccade error (difference between the saccade landing point and cue location), normalized based on target eccentricity (since larger errors are expected for more eccentric targets). There was no significant effect of the presence of visual probes on saccade error (Supplementary Fig. 6; saccade error_with probes_=0.212 ± 0.012, n=54 session-memory condition combinations; saccade error_without probes_=0.232 ± 0.009, n=65 session-memory condition combinations; with vs. without probes, p=0.106). There was no significant relationship between individual probe locations and saccade error (Pearson correlation; r=-0.012, p=0.568). There was also no significant relationship between the mean probe location, in terms of distance from the remembered location, and saccade error (Pearson correlation; r=-0.044, p=0.114). There was a significant increase in reaction times when visual probes appeared during the delay (reaction time_with probes_=209.131 ± 4.288 ms, n=54 session-memory conditions; reaction time_without probes_=185.261 ± 2.463 ms, n=65 session-memory conditions, p=0.0001 Wilcoxon rank-sum test). There was no significant relationship between individual probe locations and reaction time (Pearson correlation; r=0.029, p=0.171). However, there was a significant relationship between the mean probe location, in terms of distance from the remembered location, and reaction time (Pearson correlation; r=0.113, p<10^-4^). Overall, then, the presentation of probes slowed reaction times, and average probe locations farther from the memory location corresponded with larger reaction times, but there was no effect on memory accuracy”*

4) Figure 5 is very hard to interpret. I can't parse what's meant to be shown in panel a (what are the 4 bars?), and panel b is similarly a bit confusing. The model in principle makes sense, but it's very challenging to intuit how the tuning profiles drawn in panel b support changes in correlations. Most of the confusion I think comes from the (I believe?) fact that the inhibitory input should only be activated in presence of visual input, but that's not easy to intuit from the figure. The authors may wish to try to clarify this illustration.

We have added a schematic illustrating the RF positioning and memory conditions for which data is shown in the bar graphs summarizing our noise correlation results (Figure 6), and we have modified the model illustration (Supplemental Figure 12). You are correct that the inhibitory input is only active in the presence of both bottom-up and top-down input. We now make this explicit in the model description (page 18 of the text and Supplementary Information page 5).

Page 18 of the text:

*“one possibility is the activation of an inhibitory, decorrelating, network in the combined presence of top-down and bottom-up input.”*

Supplementary Information page 5:

*“The local inhibitory network illustrated in the figure is only active—or, alternatively, only active strongly enough to influence the excitatory network—in the presence of both bottom-up and top-down input”*

Minor:

1) Pg 21 - What color were the square targets for the MGS task? And why is the target in Fig. 1 a circle?

All the stimuli presented on the screen (fixation point, targets, and visual probes) were white circles; this was originally mis-described in one location, which we have now corrected in the Methods. (Sometimes the colors or shapes used to illustrate stimulus positioning in the figures are different for the clarity of the illustration.)

2) MGS task - a +/- 4 dva response window seems very large - how precise were the animals in their response? Were they closer to the actual target location, or to the other possible locations, which seem to be < 4 dva apart (Fig. 1c, at least for some sessions).

Mean saccade error (distance from target location) was 1.655 ± 0.108 dva with probes, and 1.744 ± 0.081 dva without probes. The additional details on the performance with and without visual probes is now included on page 4 of the Supplementary Information and the Supplementary Fig. 6.

3) For the RF mapping task (pg 20), it seems as though the responses to all probes were used. However, for the memory guided saccade task with visual probes (pg 21-22), the first probe was dropped (lines 495-496) - why was this?

We dropped the response of neurons to the first probe during the memory period to make sure there was no response contamination by any memory target offset response. We have now clarified this in the Methods, page 22:

*“The first probe from each trial was excluded from the analysis, to prevent contamination by a response to the offset of the memory target.”*

4) The authors should include a data/code availability statement (this is mentioned in the checklist, but I cannot find it in the manuscript). I encourage the authors to deposit data sufficient to reproduce the results reported in the manuscript and associated analysis code on a public repository like OSF

We now specify on page 26” *“Data and code used for analysis are available upon reasonable request.”*

5) Figs 2 & 4 - for the single-neuron examples, the authors use a shuffling procedure to evaluate significance - could they elaborate on this procedure in the methods? Is this applied to all neurons and only significant neurons included in further analyses? This does not appear to be the case, but it could be useful to clarify. Additionally, the authors could include a measure of variability in the single-neuron bar graphs - perhaps a gray band or dashed line near each bar marking the confidence interval of the shuffled 'null' distribution?

To evaluate the significance of individual sample pairs’ noise correlations, we obtained a null distribution by shuffling the trial pairings between the neurons 100 times, and calculating the noise correlation for each shuffled version; we then tested whether the actual noise correlation was significantly different from the mean of the shuffled distribution (Wilcoxon signed-rank test). We have added this information to the Methods (page 25). Significance of noise correlation changes of individual pairs was not a criteria for inclusion in any other analysis. We added a gray bar to each bar in Figs 2 and 4 (now 5) to show the confidence interval of the shuffled 'null' distribution.

6) The scatterplots in Figs 3 and 4 should be better labeled, and the remaining cases also shown (either in a separate panel or as unfilled circles). For example, at present, Fig. 3a shows, in blue, correlation coefficients for pairs of neurons with overlapping RFs and WM location in their shared peak, and in red partially-overlapping pairs of neurons with a WM location in their flanks. There are other cases, though, like overlapping pairs with WM location in flanks, partially-overlapping pairs with WM location in one or another peak, etc. I understand the two displayed conditions are the most salient of the report, but if there's a way to show the entire dataset more clearly, that would be ideal. I believe this information may be found in the heatmap in Fig 3b and the line graphs in Fig. 4d-e, but those do not show individual data points.

We have added labels to the scatterplots as requested. We have added two additional Supplementary figures presenting the scatter plots for the remaining conditions—WM at Flanks and Tails for the overlapping pairs, and WM at Peaks and Out-flanks for partially overlapping pairs, both in the absence (Sup. Fig. 4) or presence (Sup. Fig. 7) of visual probes. No significant changes in noise correlation were observed for any of these combinations. The statistics are included in the text on page 9 without probes:

*“For the pairs with overlapping RFs, remembering a location in the Flanks or Tails of the RFs had no significant effect on the noise correlation (Supplementary Fig. 4a&b, n=85 pairs, ∆correlation_Flanks_=0.002 ± 0.007, p=0.804; n=22 pairs, ∆correlation_Tails_=0.005 ± 0.015, p=0.987). For the pairs with partially-overlapping RFs, remembering a location in the peak of one RF or an Out-flanks location had no effect on the correlation between neurons (Supplementary Fig. 4c&d, n=118 pairs, ∆correlation_Peaks_=-0.0001 ± 0.004, p=0.781; n=40 pairs, ∆correlation_Out-flanks_=-0.010 ± 0.008, p=0.288). In the presence of visual probes, no significant changes in the noise correlations were observed for memory locations in the Flanks or Tails locations for overlapping pairs (Supplementary Fig. 7a&b, n=116 pairs, ∆correlation_Flanks_=-0.003 ± 0.012, p=0.559; n=141 pairs, ∆correlation_Tails_=0.001 ± 0.015, p=0.970). For partially-overlapping pairs, no significant change was observed in the Peaks or Out-flanks conditions (Supplementary Fig. 7c&d, n=57 pairs, ∆correlation_Peaks_=0.008 ± 0.019, p=0.827; n=69 pairs, ∆correlation_Out-flanks_=0.004 ± 0.014, p=0.233).”*

7) Fig. 4b-c - are the RF contours in these schematics computed from the RF mapping trials (no WM) or from the MGS task with visual probes (Fig. 4a) trials? I imagine both could be computed for these pairs to address the signal correlation point made above, and to allude to results reported previously in Merrikhi et al, 2017

These RF contours in the figure are derived from the RF mapping task. We have added a discussion of RF shifts and expansions during WM to the paper, as well as looking for a relationship between these RF changes and the changes in noise correlations (Fig. 5, page 13-16).

8) Fig. 4d-e could be more clearly labeled - if I'm understanding correctly, the x axis is the mean response to the probe given a constant WM location? If so, perhaps "geometric mean of normalized probe responses" would be an appropriate x axis label? And I'm also having trouble understanding the relationship between WM location and probe location - where is the WM location relative to the probe? Is it any of the 4 "in" locations?

The reviewer is right, “geometric mean of normalized probe responses” is the correct label for the x-axis in Fig. 4e-f and also for supplementary figures 8-9. There were more (and more tightly spaced) potential probe locations than potential WM locations-- see the grid of probe positions in Fig. 1a compared to the WM locations in Fig. 1c. In Fig. 4b the WM target is at the location indicated by the arrow-- so corresponding to just one of the 4 probe locations inside both RFs.

9) Supplementary Figs. 4 & 5 are informative, but very challenging to parse - I'd recommend adding labels to the figures above panels a, c, e indicating that these correspond to trials in which the animal is remembering in the peak, flanks, or tails of the pair, and a super-title over each figure labeling Supp Fig. 4 as showing results from overlapping and Supp Fig. 5 as showing results from partially-overlapping pairs.

Labels indicating memory position (Flanks, Tails, etc) and pair types were added.

Reviewers' comments:

Reviewer #1 (Remarks to the Author):

The authors have adequately addressed all of my concerns.

Reviewer #2 (Remarks to the Author):

The authors have made considerable effort to address my two main concerns. I appreciate their effort but have a few remaining issues concerning the approach of their control analyses:

First, to address my first question the authors analyzed the interval from 100-200 ms following micro-saccades and found a significant rate modulation for micro-saccades directed towards the WM location (pages 2-3 of Supplementary Information and Supplementary Fig 5). They also found the direction of micro-saccade was biased towards the WM location during the memory period. They then use that same interval to consider if noise correlations are significantly influenced by micro-saccade related firing rate modulations. I am largely satisfied with their control analysis, but am curious why did they restrict their analysis to either the 0-100ms or 100-200 ms interval following saccades? For example, the authors might first determine from their own data over what intervals the micro-saccades caused a significant rate modulation both before and after the micro-saccade. Are the intervals they focused on, either 0-100 or 100-200 ms, the only ones that yielded a significant modulation? If they used a sliding window of width 100 ms would they find there is a range of intervals (potentially including a couple hundred milliseconds before the micro-saccade, aka, presaccadic attention) that show firing rate modulations? Once the range that is significantly modulated is determined, the most conservative analysis would be to remove that entire range from the noise correlation analysis, not only a smaller part of it, and demonstrate the results still stand. The concern here is that the modulation may be broader, ranging over an epoch of 200-300 ms in duration or even prior to the micro-saccade, and that removing either 0-100 ms or 100-200 ms alone does not alter the effects because they are smaller part of the total impact of the micro-saccade. Also note, the rate modulation could be significantly negative rather than positive, but either direction of rate modulation could still impact the noise correlations, so all intervals significantly modulated should be excluded.

They address my second concern about the effect of stimulus history for probe stimuli prior to the current probe on noise correlations by performing an analysis on the data in two halves, the first in which the previous probe evoked a larger firing rate for the neuron under study and the second in which it evoked a weaker firing rate (Supplementary information pages 4-5 and Supplementary Figure 10). First they confirmed the previous probe did cause a change in firing rate for the neuron to the current probe which is consistent with an adaptation effect (weaker response if the previous probe was a stronger stimulus). The authors then find “There is no difference between noise correlation of neurons during weak and strong trials”; however, the key question here is not if the noise correlation differs between weak and strong trials but whether that by partitioning the trials this way it removes a key source of “shared” variation in firing rates that would reduce noise
correlations. In short, is the noise correlation reduced when you analyze only the weak or strong trials separately? If so this demonstrates that factor may have contributed significantly to the noise correlations and possibly the main findings. Then the key question is do the main findings of the study remain even when this source of variance is removed by partitioning the trials into these halves? The third reviewer is raising a similar concern in their discussion about “signal” versus “noise” correlations. The history dependence of the firing here could be considered “signal” correlations, as it is driven by the stimulus history whereas “noise” correlations is typically referring to what are thought to be identically repeated stimuli. This is an important concern to address for the interpretation of these results.

Reviewer #3 (Remarks to the Author):

I appreciate the authors’ careful attention to my feedback in their revision. I think the result is a very comprehensive, interesting, and important report describing how the relationship between visual input and top-down feedback impact noise correlations as a function of recorded neuron RF properties. I also find the microsaccade analysis very interesting. The manuscript, with maybe one small edit to improve readability of the supplement, will make an excellent contribution to Nature Communications.

Supp Info, pg 6 - “A critical difference between their task and ours is the behavioral relevance of the visual stimulus—in their task, an explicit comparison between visual stimuli at different locations is the basis for task performance, and the observed increases and decreases in RF are consistent with improving the neural basis for that comparison.” – it’s unclear what increases/decreases in RF means – do the authors mean to say increases/decreases in noise correlations? And what do they mean by j”an explicit comparison between visual stimuli at different locations” – is this referring to an attended/unattended stimulus? Comparison operation between both stimuli? This paragraph could be slightly clarified to improve readability.

Reviewers' comments:

Reviewer #1 (Remarks to the Author):

The authors have adequately addressed all of my concerns.

We thank the reviewer.

Reviewer #2 (Remarks to the Author):

The authors have made considerable effort to address my two main concerns. I appreciate their effort but have a few remaining issues concerning the approach of their control analyses:

First, to address my first question the authors analyzed the interval from 100-200 ms following micro-saccades and found a significant rate modulation for micro-saccades directed towards the WM location (pages 2-3 of Supplementary Information and Supplementary Fig 5). They also found the direction of micro-saccade was biased towards the WM location during the memory period. They then use that same interval to consider if noise correlations are significantly influenced by micro-saccade related firing rate modulations. I am largely satisfied with their control analysis, but am curious why did they restrict their analysis to either the 0-100ms or 100-200 ms interval following saccades? For example, the authors might first determine from their own data over what intervals the micro-saccades caused a significant rate modulation both before and after the micro-saccade. Are the intervals they focused on, either 0-100 or 100-200 ms, the only ones that yielded a significant modulation? If they used a sliding window of width 100 ms would they find there is a range of intervals (potentially including a couple hundred milliseconds before the micro-saccade, aka, presaccadic attention) that show firing rate modulations? Once the range that is significantly modulated is determined, the most conservative analysis would be to remove that entire range from the noise correlation analysis, not only a smaller part of it, and demonstrate the results still stand. The concern here is that the modulation may be broader, ranging over an epoch of 200-300 ms in duration or even prior to the micro-saccade, and that removing either 0-100 ms or 100-200 ms alone does not alter the effects because they are smaller part of the total impact of the micro-saccade. Also note, the rate modulation could be significantly negative rather than positive, but either direction of rate modulation could still impact the noise correlations, so all intervals significantly modulated should be excluded.

We chose the 100-200 ms interval after microsaccade because this time window was recently reported for microsaccade-associated firing rate modulations (Lowet et al., Neuron 2018). However we thank the reviewer for this suggestion. As suggested, we performed a comprehensive analysis exploring the response of neurons from 450 ms before each microsaccde to 400 ms after using sliding windows with different widths (ranging from 50 to 200 ms) to identify the specific time interval associated with these firing changes. We found that during the memory period, the firing rate of neurons significantly increased after each microsaccade. The maximum rate modulation was observed for a window width of 150 ms starting from 90 ms after the microsaccade. Then we excluded the period 90-240ms after any microsaccades from the noise correlation analysis. We still found an increase in noise correlations during memory at the Peak location for overlapping pairs and a decrease in noise correlation during memory at the In-flank location for partially overlapping pairs. We have added these results to the Supplementary information, pages 2-3 and the Supplementary Fig.5b-f:

*“To determine whether the occurrence of microsaccades caused any significant changes in the firing rate of MT neurons, and also to identify the specific time interval either before or after each microsaccade associated with these firing changes (if they occur), we measured a rate modulation index using sliding windows with different widths (ranging from 50 to 200 ms), starting at different times relative to the onset of microsaccades (ranging from 450 ms before each microsaccade onset to 400 ms after). The rate modulation was calculated as follows: rate modulation index=(FR_sliding window_ – FR_baseline_)/ (FR_sliding window_+ FR_baseline_). The baseline firing rate is the firing rate during a time interval with the width of sliding window, starting 500 ms before each microsaccade. We found that during the memory period, the firing rate of neurons significantly increased after each microsaccade. The maximum rate modulation was observed for a window width of 150 ms starting from 90 ms after the microsaccade (n=425 neuron-location combinations, rate modulation=0.088 ± 0.019, p=0.00003, Supplementary Fig. 5b). Specifically, Supplementary Fig.5c shows the rate modulation of MT neurons measured with a sliding window with a width of 150 ms at different times relative to microsaccade onset. The bold portion shows the time period with significant rate modulation after microsaccade onset (p<0.05). Again, the maximum rate modulation is observed 90ms after the onset of microsaccades. This time interval is very similar to the time window reported for previous microsaccade-associated firing rate modulations^13^. We then measured the rate modulation using this time interval (90-240 ms after each microsaccade, compared to a baseline 150-0 ms before each microsaccade in which we found no significant rate modulation (Supplementary Fig.5c, n=425 neuron-location combinations, rate modulation=0.025 ± 0.020, p=0.221)) during the memory and fixation periods. During the memory period the overall rate modulation was significantly increased after microsaccades (n=425 neuron-location combinations, rate modulation=0.029 ± 0.010, p=0.037). We also found that microsaccades toward remembered locations are associated with greater rate modulation (Supplementary Fig. 5d, green plot; the negative values on x-axis indicates microsaccades toward a remembered location and the bold portion indicates significant rate modulation (p<0.05), Pearson correlation; r=-0.06, p=0.025). During fixation there was no overall rate modulation 90-240ms after microsaccades, and also no relationship between the direction of microsaccades and rate modulation (n=425 neuron-location combinations, rate modulation=0.006 ± 0.014, p=0.308, Supplementary Fig. 5d, gray plot, Pearson correlation; r=0.087, p=0.081).*

*Finally, we examined the relationship between microsaccades and noise correlations. During the fixation period, we see no change in noise correlation values before vs. after microsaccades (n=350 pairs, ∆correlation_after-before_=-0.014 ± 0.010, p=0.667; Supplementary Fig. 5e); nor was there any relationship between microshift direction and changes in noise correlation (Pearson correlation; r=-0.016, p=0.774). Then* *we asked whether the observed changes in noise correlation based on memory location were still significant when excluding the time period in which microsaccade-driven rate modulation occurred. When we exclude the period 90-240ms after any microsaccades from the noise correlation analysis, we still see an increase in noise correlations during memory at the Peak location for overlapping pairs (n=43 pairs; ∆correlation_Peak_=0.025 ± 0.010, p=0.016; Supplementary Fig. 5f blue), and a decrease in noise correlation during memory at the In-flank location for partially overlapping pairs (n=103 pairs, ∆correlation_In-flank_=-0.017 ± 0.005, p=0.001; Supplementary Fig. 5f red). Altogether, these results demonstrate that microsaccades are biased by WM, but do not drive the changes in noise correlations during WM.”*

They address my second concern about the effect of stimulus history for probe stimuli prior to the current probe on noise correlations by performing an analysis on the data in two halves, the first in which the previous probe evoked a larger firing rate for the neuron under study and the second in which it evoked a weaker firing rate (Supplementary information pages 4-5 and Supplementary Figure 10). First they confirmed the previous probe did cause a change in firing rate for the neuron to the current probe which is consistent with an adaptation effect (weaker response if the previous probe was a stronger stimulus). The authors then find “There is no difference between noise correlation of neurons during weak and strong trials”; however, the key question here is not if the noise correlation differs between weak and strong trials but whether that by partitioning the trials this way it removes a key source of “shared” variation in firing rates that would reduce noise
correlations. In short, is the noise correlation reduced when you analyze only the weak or strong trials separately? If so this demonstrates that factor may have contributed significantly to the noise correlations and possibly the main findings. Then the key question is do the main findings of the study remain even when this source of variance is removed by partitioning the trials into these halves? The third reviewer is raising a similar concern in their discussion about “signal” versus “noise” correlations. The history dependence of the firing here could be considered “signal” correlations, as it is driven by the stimulus history whereas “noise” correlations is typically referring to what are thought to be identically repeated stimuli. This is an important concern to address for the interpretation of these results.

We added this control as suggested and confirm that there is no significant change of noise correlation if we analyze either the weak or strong trials separately. We did not originally add this analysis just to avoid splitting the data into this many conditions. We agree with the reviewer that it is at least worth reporting that we do not observe differences between weak and strong trials. We have added these results to the Supplementary information, page 5 and the Supplementary Fig.10e-f:

*“We also confirmed that there is no siginificant difference between noise correlation of pairs if we consider either strong (∆correlation_strong-all_=-0.004 ± 0.011, p=0.935, n=121 pairs; Supplementary Fig. 10e) or weak (∆correlation_weak-all_=0.002 ± 0.008, p=0.753, n=121 pairs; Supplementary Fig. 10f) trials* *separately*.”

We also added another control to examine how partitioning the trials into weak and strong trials changes the main findings for the overlapping and partially overlapping pairs. We have added these results to the Supplementary information, page 5:

*“Finally we examined how partitioning the trials into weak and strong trials changes the main findings for the overlapping and partially overlapping pairs shown in figure 4d. We confirmed that there is no significant difference between the noise correlation changes for the Peak location of overlapping pairs if we consider either weak or strong trials (∆correlation_weak-strong_=0.067 ± 0.148, p=0.840; ∆correlation_weak-all_=0.075 ± 0.077, p=0.193; ∆correlation_strong-all_=-0.051 ± 0.140, p=0.839; n=17 overlapping pairs). We also did not find a significant difference between the noise correlation changes for the In-flank location of partially overlapping pairs when we analyze either weak or strong trials (∆correlation_weak-strong_=0.022 ± 0.134, p=0.961; ∆correlation_weak-all_=-0.006 ± 0.035, p=0.596; ∆correlation_strong-all_=-0.032 ± 0.133, p=0.731; n=56 partially overlapping pairs).This verifies that the adaptation and stimulus history are not contributing to the observed noise correlation changes.”*

Reviewer #3 (Remarks to the Author):

I appreciate the authors’ careful attention to my feedback in their revision. I think the result is a very comprehensive, interesting, and important report describing how the relationship between visual input and top-down feedback impact noise correlations as a function of recorded neuron RF properties. I also find the microsaccade analysis very interesting. The manuscript, with maybe one small edit to improve readability of the supplement, will make an excellent contribution to Nature Communications.

Supp Info, pg 6 - “A critical difference between their task and ours is the behavioral relevance of the visual stimulus—in their task, an explicit comparison between visual stimuli at different locations is the basis for task performance, and the observed increases and decreases in RF are consistent with improving the neural basis for that comparison.” – it’s unclear what increases/decreases in RF means – do the authors mean to say increases/decreases in noise correlations? And what do they mean by j”an explicit comparison between visual stimuli at different locations” – is this referring to an attended/unattended stimulus? Comparison operation between both stimuli? This paragraph could be slightly clarified to improve readability.

We thank the reviewer for noting this error—the sentence should read*, “the observed increases and decreases in noise correlations”* instead of RFs. Per the reviewer’s suggestion we have clarified our description of the task used by Ruff and Cohen to read *“an explicit comparison of the contrast values of visual stimuli at different locations”.*

Reviewers' comments:

Reviewer #2 (Remarks to the Author):

The authors have adequately addressed all of my concerns. I appreciate their effort.

Reviewers' comments:

Reviewer #2 (Remarks to the Author):

The authors have adequately addressed all of my concerns. I appreciate their effort.

We thank the reviewer.
